# Decorrelation of Neural-Network Activity by Inhibitory Feedback

**DOI:** 10.1371/journal.pcbi.1002596

**Published:** 2012-08-02

**Authors:** Tom Tetzlaff, Moritz Helias, Gaute T. Einevoll, Markus Diesmann

**Affiliations:** 1 Institute of Neuroscience and Medicine (INM-6), Computational and Systems Neuroscience, Research Center Jülich, Jülich, Germany; 2 CIGENE, Department of Mathematical Sciences and Technology, Norwegian University of Life Sciences, Ås, Norway; 3 RIKEN Brain Science Institute and Brain and Neural Systems Team, RIKEN Computational Science Research Program, Wako, Japan; 4 Medical Faculty, RWTH Aachen University, Aachen, Germany; Université Paris Descartes, France

## Abstract

Correlations in spike-train ensembles can seriously impair the encoding of information by their spatio-temporal structure. An inevitable source of correlation in finite neural networks is common presynaptic input to pairs of neurons. Recent studies demonstrate that spike correlations in recurrent neural networks are considerably smaller than expected based on the amount of shared presynaptic input. Here, we explain this observation by means of a linear network model and simulations of networks of leaky integrate-and-fire neurons. We show that inhibitory feedback efficiently suppresses pairwise correlations and, hence, population-rate fluctuations, thereby assigning inhibitory neurons the new role of active decorrelation. We quantify this decorrelation by comparing the responses of the intact recurrent network (feedback system) and systems where the statistics of the feedback channel is perturbed (feedforward system). Manipulations of the feedback statistics can lead to a significant increase in the power and coherence of the population response. In particular, neglecting correlations within the ensemble of feedback channels or between the external stimulus and the feedback amplifies population-rate fluctuations by orders of magnitude. The fluctuation suppression in homogeneous inhibitory networks is explained by a negative feedback loop in the one-dimensional dynamics of the compound activity. Similarly, a change of coordinates exposes an effective negative feedback loop in the compound dynamics of stable excitatory-inhibitory networks. The suppression of input correlations in finite networks is explained by the population averaged correlations in the linear network model: In purely inhibitory networks, shared-input correlations are canceled by negative spike-train correlations. In excitatory-inhibitory networks, spike-train correlations are typically positive. Here, the suppression of input correlations is not a result of the mere existence of correlations between excitatory (E) and inhibitory (I) neurons, but a consequence of a particular structure of correlations among the three possible pairings (EE, EI, II).

## Author Summary

The spatio-temporal activity pattern generated by a recurrent neuronal network can provide a rich dynamical basis which allows readout neurons to generate a variety of responses by tuning the synaptic weights of their inputs. The repertoire of possible responses and the response reliability become maximal if the spike trains of individual neurons are uncorrelated. Spike-train correlations in cortical networks can indeed be very small, even for neighboring neurons. This seems to be at odds with the finding that neighboring neurons receive a considerable fraction of inputs from identical presynaptic sources constituting an inevitable source of correlation. In this article, we show that inhibitory feedback, abundant in biological neuronal networks, actively suppresses correlations. The mechanism is generic: It does not depend on the details of the network nodes and decorrelates networks composed of excitatory and inhibitory neurons as well as purely inhibitory networks. For the case of the leaky integrate-and-fire model, we derive the correlation structure analytically. The new toolbox of formal linearization and a basis transformation exposing the feedback component is applicable to a range of biological systems. We confirm our analytical results by direct simulations.

## Introduction

Neurons generate signals by weighting and combining input spike trains from presynaptic neuron populations. The number of possible signals which can be read out this way from a given spike-train ensemble is maximal if these spike trains span an orthogonal basis, i.e. if they are uncorrelated [1]. If they are correlated, the amount of information which can be encoded in the spatio-temporal structure of these spike trains is limited. In addition, correlations impair the ability of readout neurons to decode information reliably in the presence of noise. This is often discussed in the context of *rate coding*: for 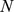 uncorrelated spike trains, the signal-to-noise ratio of the compound spike-count signal can be enhanced by increasing the population size 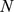. In the presence of correlations, however, the signal-to-noise ratio is bounded [2], [3]. The same reasoning holds for any other linear combination of spike trains, also for those where exact spike timing matters (for example for the coding scheme presented in [4]). Thus, the robustness of neuronal responses against noise critically depends on the level of correlated activity within the presynaptic neuron population.

Several studies suggested that correlated neural activity could be beneficial for information processing: Spike-train correlations can modulate the gain of postsynaptic neurons and thereby constitute a gating mechanism (for a review, see [4]). Coherent spiking activity might serve as a means to bind elementary representations into more complex objects [5], [6]. Information represented by correlated firing can be reliably sustained and propagated through feedforward subnetworks (‘synfire chains’; [7], [8]). Whether correlated firing has to be considered favorable or not largely depends on the underlying hypothesis, the type of correlation (e.g. the time scale or the affected frequency band) or which subpopulations of neurons are involved. Most ideas suggesting a functional benefit of correlated activity rely on the existence of an asynchronous ‘ground state’. Spontaneously emerging correlations, i.e. correlations which are not triggered by internal or external events, would impose a serious challenge to many of these hypotheses. Functionally relevant synfire activity, for example, cannot be guaranteed in the presence of correlated background input from the embedding network [9]. It is therefore–from several perspectives–important to understand the origin of uncorrelated activity in neural networks.

It has recently been shown that spike trains of neighboring cortical neurons can indeed be uncorrelated [10]. Similar results have been obtained in several theoretical studies [11]–[17]. From an anatomical point of view, this observation is puzzling: in general, neurons in finite networks share a certain fraction of their presynaptic sources. In particular for neighboring neurons, the overlap between presynaptic neuron populations is expected to be substantial. This feedforward picture suggests that such presynaptic overlap gives rise to correlated synaptic input and, in turn, to correlated response spike trains.

A number of theoretical studies showed that shared-input correlations are only weakly transferred to the output side as a consequence of the nonlinearity of the spike-generation dynamics [15], [18]–[21]. Unreliable spike transmission due to synaptic failure can further suppress the correlation gain [22]. In [9], we demonstrated that spike-train correlations in finite-size recurrent networks are even smaller than predicted by the low correlation gain of pairs of neurons with nonlinear spike-generation dynamics. We concluded that this suppression of correlations must be a result of the recurrent network dynamics. In this article, we compare correlations observed in feedforward networks to correlations measured in systems with an intact feedback loop. We refer to the reduction of correlations in the presence of feedback as “decorrelation”. Different mechanisms underlying such a dynamical decorrelation have been suggested in the recent past. Asynchronous states in recurrent neural networks are often attributed to chaotic dynamics [23], [24]. In fact, networks of nonlinear units with random connectivity and balanced excitation and inhibition typically exhibit chaos [11], [25]. The high sensitivity to noise may however question the functional relevance of such systems ([26], [27]; cf., however, [28]). [29] and [27] demonstrated that asynchronous irregular firing can also emerge in networks with stable dynamics. Employing an analytical framework of correlations in recurrent networks of binary neurons [30], the balance of excitation and inhibition has recently been proposed as another decorrelation mechanism [17]: In large networks, fluctuations of excitation and inhibition are in phase. Positive correlations between excitatory and inhibitory input spike trains lead to a negative component in the net input correlation which can compensate positive correlations caused by shared input.

In the present study, we demonstrate that dynamical decorrelation is a fundamental phenomenon in recurrent systems with negative feedback. We show that negative feedback alone is sufficient to efficiently suppress correlations. Even in purely inhibitory networks, shared-input correlations are compensated by feedback. A balance of excitation and inhibition is thus not required. The underlying mechanism can be understood by means of a simple linear model. This simplifies the theory and helps to gain intuition, but it also confirms that low correlations can emerge in recurrent networks with stable, non-chaotic dynamics.

The suppression of pairwise spike-train correlations by inhibitory feedback is reflected in a reduction of population-rate fluctuations. The main effect described in this article can therefore be understood by studying the dynamics of the macroscopic population activity. This approach leads to a simple mathematical description and emphasizes that the described decorrelation mechanism is a general phenomenon which may occur not only in neural networks but also in other (biological) systems with inhibitory feedback. In **“** **Results**: **Suppression of population-rate fluctuations in LIF networks”**, we first illustrate the decorrelation effect for random networks of 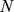 leaky integrate-and-fire (LIF) neurons with inhibitory or excitatory-inhibitory coupling. By means of simulations, we show that low-frequency spike-train correlations, and, hence, population-rate fluctuations are substantially smaller than expected given the amount of shared input. As shown in the subsequent section, the **“Suppression of population-activity fluctuations by negative feedback”** can readily be understood in the framework of a simple one-dimensional linear model with negative feedback. In **“** **Results** **: Population-activity fluctuations in excitatory-inhibitory networks”**, we extend this to a two-population system with excitatory-inhibitory coupling. Here, a simple coordinate transform exposes the inherent negative feedback loop as the underlying cause of the fluctuation suppression in inhibition-dominated networks. The population-rate models of the inhibitory and the excitatory-inhibitory network are sufficient to understand the basic mechanism underlying the decorrelation. They do, however, not describe how feedback in cortical networks affects the detailed structure of pairwise correlations. In **“** **Results** **: Population averaged correlations in cortical networks”**, we therefore compute self-consistent population averaged correlations for a random network of 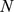 linear excitatory and inhibitory neurons. By determining the parameters of the linear network analytically from the LIF model, we show that the predictions of the linear model are—for a wide and realistic range of parameters—in excellent agreement with the results of the LIF network model. In **“** **Results** **: Effect of feedback manipulations”**, we demonstrate that the active decorrelation in random LIF networks relies on the feedback of the (sub)population averaged activity but not on the precise microscopic structure of the feedback signal. In the **“** **Discussion** **”**, we put the consequences of this work into a broader context and point out limitations and possible extensions of the presented theory. The **“** **Methods** **”** contain details on the LIF network model, the derivation of the linear model from the LIF dynamics and the derivation of population-rate spectra and population averaged correlations in the framework of the linear model. This section is meant as a supplement; the basic ideas and the main results can be extracted from the **“** **Results** **”**.

## Results

**Figure 1:**
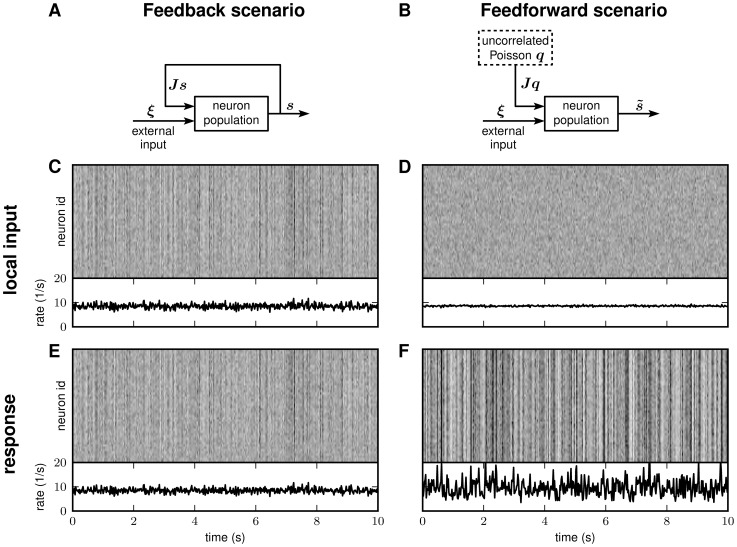
Spiking activity in excitatory-inhibitory LIF networks with intact (left column; *feedback scenario*) and opened feedback loop (right column; *feedforward scenario*). **A,B**: Network sketches for the feedback (A) and feedforward scenario (B). **C,D**: Spiking activity (top panels) and population averaged firing rate (bottom panels) of the local presynaptic populations. **E,F**: Response spiking activity (top panels) and population averaged response rate (bottom panels). In the top panels of C–F, each pixel depicts the number of spikes (gray coded) of a subpopulation of 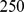 neurons in a 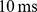 time interval. In both the feedback and the feedforward scenario, the neuron population 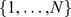 is driven by the same realization 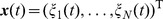 of an uncorrelated white-noise ensemble; local input is fed to the population through the same connectivity matrix 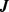. The in-degrees, the synaptic weights and the shared-input statistics are thus exactly identical in the two scenarios. In the feedback case (A), local presynaptic spike-trains are provided by the network's response 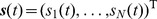, i.e. the pre- (C) and postsynaptic spike-train ensembles (E) are identical. In the feedforward scenario (B), the local presynaptic spike-train population is replaced by an ensemble of 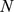 independent realizations 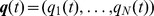 of a Poisson point process (D). Its rate is identical to the time- and population-averaged firing rate in the feedback case. See Table 1 and Table 2 for details on network models and parameters.

In a recurrent neural network of size 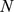, each neuron 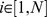 receives in general inputs from two different types of sources: External inputs 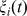 representing the sum of afferents from other brain areas, and local inputs resulting from the recurrent connectivity within the network. Depending on their origin, external inputs 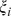 and 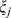 to different neurons 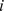 and 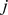 can be correlated or not. Throughout this manuscript, we ignore correlations between these external sources, thereby ensuring that correlations within the network activity arise from the local connectivity alone and are not imposed by external inputs [17]. The local inputs feed the network's spiking activity 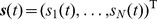 back to the network (we refer to spike train 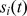, the 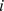 th component of the column vector 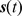 [the superscript “
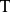
” denotes the transpose], as a sum over delta-functions centered at the spike times 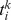: 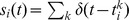; the abstract quantity ‘spike train’ can be considered as being derived from the observable quantity ‘spike count’ 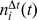, the number of spikes occurring in the time interval 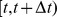, by taking the limit 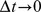: 
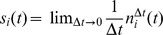
). The structure and weighting of this feedback can be described by the network's connectivity matrix 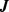 (see Fig. 1 A). In a finite network, the local connectivity typically gives rise to overlapping presynaptic populations: in a random (Erdös-Rényi) network with connection probability 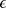, for example, each pair of postsynaptic neurons shares, on average, 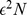 presynaptic sources. For a network size of, say, 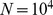 and a connection probability 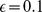, this corresponds to a fairly large number of 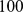 identical inputs. For other network structures, the amount of shared input may be smaller or larger. Due to this presynaptic overlap, each pair of neurons receives, to some extent, correlated input (even if the external inputs are uncorrelated). One might therefore expect that the network responses 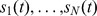 are correlated as well. In this article, we show that, in the presence of negative feedback, the effect of shared input caused by the structure of the network is compensated by its recurrent dynamics.

### Suppression of population-rate fluctuations in LIF networks

To illustrate the effect of shared input and its suppression by the recurrent dynamics, we compare the spike response 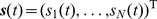 of a recurrent random network (*feedback scenario*; Fig. 1 A,C,E) of 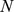 LIF neurons to the case where the feedback is cut and replaced by a spike-train ensemble 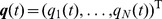, modeled by 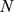 independent realizations of a stationary Poisson point process (*feedforward scenario*; Fig. 1 B,D,F). The rate of this Poisson process is identical to the time and population averaged firing rate in the intact recurrent system. In both the feedback and the feedforward case, the (local) presynaptic spike trains are fed to the postsynaptic population according to the same connectivity matrix 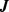. Therefore, not only the in-degrees and the synaptic weights but also the shared-input statistics are exactly identical.

For realistic size 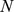 and connectivity 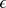, asynchronous states of random neural networks [12], [31] exhibit spike-train correlations which are small but not zero (compare raster displays in Fig. 1 C and D; see also [15]). Although the presynaptic spike trains are, by construction, independent in the feedforward case (Fig. 1 D), the resulting response correlations, and, hence, the population-rate fluctuations, are substantially stronger than those observed in the feedback scenario (compare Fig. 1 F and E). In other words: A theory which is exclusively based on the amount of shared input but neglects the details of the presynaptic spike-train statistics can significantly overestimate correlations and population-rate fluctuations in recurrent neural networks.

**Figure 2:**
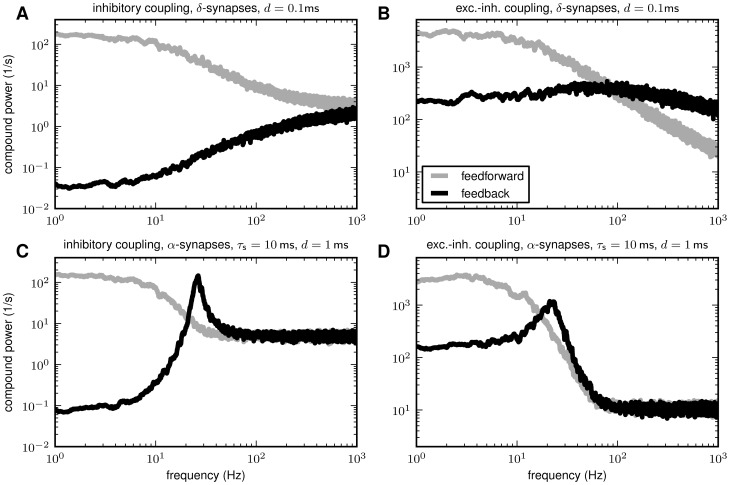
Suppression of low-frequency fluctuations in recurrent LIF networks with purely inhibitory (A, C) and mixed excitatory-inhibitory coupling (B, D) for instantaneous synapses with delay 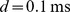 (A, B) and low-pass synapses with 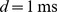 (C, D). Power-spectra 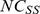 of population rates 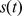 for the feedback (black) and the feedforward case (gray; cf. Fig. 1). See Table 1 and Table 2 for details on network models and parameters. In C and D, local synaptic inputs are modeled as currents 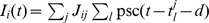 with 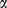-function shaped kernel 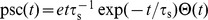 with time constant 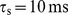 (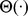 denotes Heaviside function). (Excitatory) Synaptic weights are set to 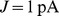 (see Table 1 for details). Simulation time 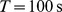. Single-trial spectra smoothed by moving average (frame size 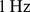).

The same effect can be observed in LIF networks with both purely inhibitory and mixed excitatory-inhibitory coupling (Fig. 2). To demonstrate this quantitatively, we focus on the fluctuations of the population averaged activity 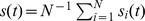. Its power-spectrum (or auto-correlation, in the time domain)


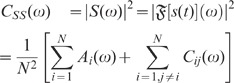
(1)

is determined both by the power-spectra (auto-correlations) 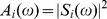 of the individual spike trains and the cross-spectra (cross-correlations) 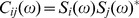 (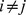) of pairs of spike trains (throughout the article, we use capital letters to represent quantities in frequency [Fourier] space; 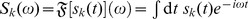 represents the Fourier transform of the spike train 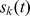). We observe that the spike-train power-spectra 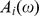 (and auto-correlations) are barely distinguishable in the feedback and in the feedforward case (not shown here; the main features of the spike-train auto-correlation are determined by the average single-neuron firing rate and the refractory mechanism; both are identical in the feedback and the feedforward scenario). The differences in the population-rate spectra 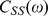 are therefore essentially due to differences in the spike-train cross-spectra 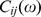. In other words, the fluctuations in the population activity serve as a measure of pairwise spike-train correlations [32]: small (large) population averaged spike-train correlations are accompanied by small (large) fluctuations in the population rate (see lower panels in Fig. 1 C–F). The power-spectra 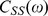 of the population averaged activity reveal a feedback-induced suppression of the population-rate variance at low frequencies up to several tens of Hertz. For the examples shown in Fig. 2, this suppression spans more than three orders of magnitude for the inhibitory and more than one order of magnitude for the excitatory-inhibitory network.

The suppression of low-frequency fluctuations does not critically depend on the details of the network model. As shown in Fig. 2, it can, for example, be observed for both networks with zero rise-time synapses (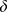-shaped synaptic currents) and short delays and for networks with delayed low-pass filtering synapses (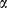-shaped synaptic currents). In the latter case, the suppression of fluctuations is slightly more restricted to lower frequencies (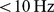). Here, the fluctuation suppression is however similarly pronounced as in networks with instantaneous synapses.

In Fig. 2 C,D, the power-spectra of the population activity converge to the mean firing rate at high frequencies. This indicates that the spike trains are uncorrelated on short time scales. For instantaneous 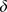-synapses, neurons exhibit an immediate response to excitatory input spikes [33], [34]. This fast response causes spike-train correlations on short time scales. Hence, the compound power at high frequencies is increased. In a recurrent system, this effect is amplified by reverberating simultaneous excitatory spikes. Therefore, the high-frequency power of the compound activity is larger in the feedback case (Fig. 2 B). Note that this high-frequency effect is absent in networks with more realistic low-pass filtering synapses (Fig. 2 C,D) and in purely inhibitory networks (Fig. 2 A).

Synaptic delays and slow synapses can promote oscillatory modes in certain frequency bands [12], [31], thereby leading to peaks in the population-rate spectra in the feedback scenario which exceed the power in the feedforward case (see peaks at 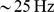 in Fig. 2 C,D). Note that, in the feedforward case, the local input was replaced by a stationary Poisson process, whereas in the recurrent network (feedback case) the presynaptic spike trains exhibit oscillatory modes. By replacing the feedback by an inhomogeneous Poisson process with a time dependent intensity which is identical to the population rate in the recurrent network, we found that these oscillatory modes are neither suppressed nor amplified by the recurrent dynamics, i.e. the peaks in the resulting power-spectra have the same amplitude in the feedback and in the feedforward case (data not shown here). At low frequencies, however, the results are identical to those obtained by replacing the feedback by a homogeneous Poisson process (i.e. to those shown in Fig. 2; see **“** **Results** **: Effect of feedback manipulations”**). In the present study, we mainly focus on these low-frequency effects.

The observation that the suppression of low-frequency fluctuations is particularly pronounced in networks with purely inhibitory coupling indicates that inhibitory feedback may play a key role for the underlying mechanism. In the following subsection, we demonstrate by means of a one-dimensional linear population model that, indeed, negative feedback alone leads to an efficient fluctuation suppression.

### Suppression of population-activity fluctuations by negative feedback

Average pairwise correlations can be extracted from the spectrum (1) of the compound activity, provided the single spike-train statistics (auto-correlations) is known (see previous section). As the single spike-train statistics is identical in the feedback and in the feedforward scenario, the mechanism underlying the decorrelation in recurrent networks can be understood by studying the dynamics of the population averaged activity. In this and in the next subsection, we consider the linearized dynamics of random networks composed of homogeneous subpopulations of LIF neurons. The high-dimensional dynamics of such systems can be reduced to low-dimensional models describing the dynamics of the compound activity (for details, see **“** **Methods** **: Linearized network model”**). Note that this reduction is exact for networks with homogeneous out-degree (number of outgoing connections). For the networks studied here (random networks with homogeneous in-degree), it serves as a sufficient approximation (in a network of size 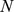 where each connection is randomly and independently realized with probability 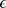 [Erdös-Rényi graph], the [binomial] in- and out-degree distributions become very narrow for large 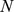 [relative to the mean in/out-degree]; both in- and out-degree are therefore approximately constant across the population of neurons). In this subsection, we first study networks with purely inhibitory coupling. In **“** **Results** **: Population-activity fluctuations in excitatory-inhibitory networks”**, we investigate the effect of mixed excitatory-inhibitory connectivity.

**Figure 3:**
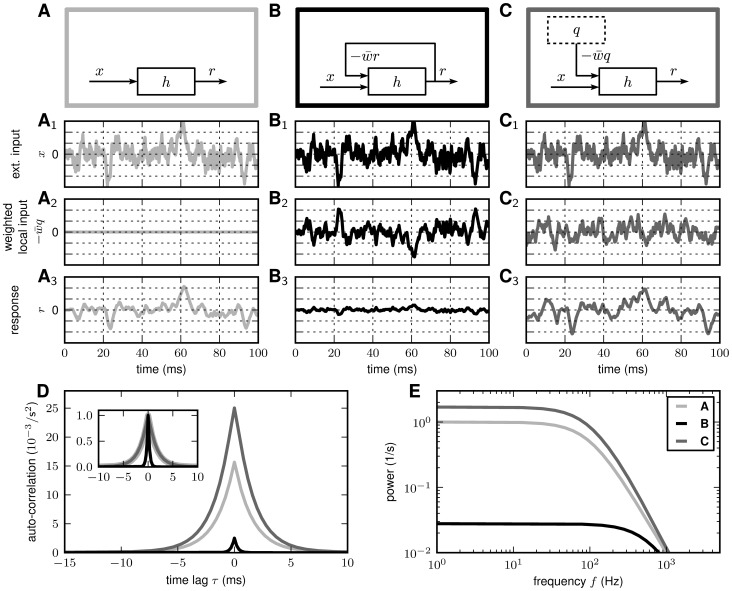
Partial canceling of fluctuations in a linear system by inhibitory feedback. Response 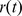 of a linear system with impulse response 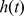 (1st-order low-pass, cutoff frequency 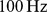) to Gaussian white noise input 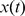 with amplitude 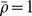 for three local-input scenarios. **A** (light gray): No feedback (local input 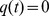). **B** (black): Negative feedback (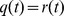) with strength 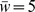. The fluctuations of the weighted local input 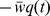 (B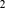) are anticorrelated to the external drive 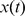 (B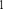). **C** (dark gray): Feedback in B is replaced by uncorrelated feedforward input 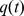 with the same auto-statistics as the response 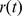 in B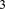. The local input 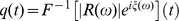 is constructed by assigning a random phase 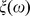 to each Fourier component 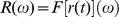 of the response in B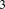. Fluctuations in C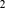 and C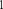 are uncorrelated. **A**, **B**, **C**: Network sketches. **A** 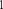, **B** 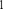, **C** 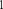: External input 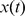. **A** 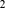, **B** 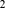, **C** 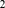: Weighted local input 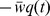. **A** 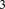, **B** 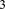, **C** 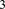: Responses 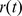. **D**, **E**: Response auto-correlation functions (D) and power-spectra (E) for the three cases shown in A,B,C (same gray coding as in A,B,C; inset in D: normalized auto-correlations).

Consider a random network of 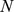 identical neurons with connection probability 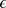. Each neuron 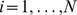 receives 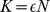 randomly chosen inputs from the local network with synaptic weights 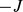. In addition, the neurons are driven by external uncorrelated Gaussian white noise 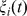 with amplitude 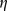, i.e. 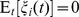 and 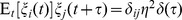. For small input fluctuations, the network dynamics can be linearized. This linearization is based on the averaged response of a single neuron to an incoming spike and describes the activity of an individual neuron 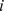 by an abstract fluctuating quantity 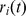 which is defined such that within the linear approximation its auto- and cross-correlations fulfill the same linearized equation as the spiking model in the low-frequency limit. Consequently, also the low-frequency fluctuations of the population spike rate are captured correctly by the reduced model up to linear order. This approach is equivalent to the treatment of finite-size fluctuations in spiking networks (see, e.g., [31]). For details, see **“** **Methods** **: Linearized network model”**. For large 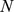, the population averaged activity 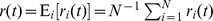 can hence be described by a one-dimensional linear system


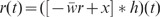
(2)

with linear kernel 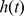, effective coupling strength 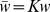 and the population averaged noise 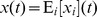 (see **“** **Methods** **: Linearized network model”** and Fig. 3 B). The coupling strength 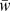 represents the integrated linear response of the neuron population to a small perturbation in the input rate of a single presynaptic neuron. For a population of LIF neurons, its relation to the synaptic weight 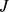 (PSP amplitude) is derived in **“** **Methods** **: Linearized network model”** and **“** **Methods** **: Response kernel of the LIF model”**. The normalized kernel 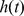 (with 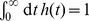) captures the time course of the linear response. It is determined by the single-neuron properties (e.g. the spike-initiation dynamics [35], [36]), the properties of the synapses (e.g. synaptic weights and time constants [37], [38]) and the properties of the input (e.g. excitatory vs. inhibitory input [39]). For many real and model neurons, the linear population-rate response exhibits low-pass characteristics [13], [34]–[46]. For illustration (Fig. 3), we consider a 1st-order low-pass filter, i.e. an exponential impulse response 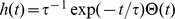 with time constant 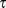 (cutoff frequency 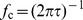; see Fig. 3 A, light gray curve in E). The results of our analysis are however independent of the choice of the kernel 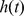. The auto-correlation 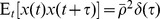 of the external noise is parametrized by the effective noise amplitude 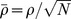.

Given the simplified description (2), the suppression of response fluctuations by negative feedback can be understood intuitively: Consider first the case where the neurons in the local network are unconnected (Fig. 3 A; no feedback, 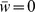). Here, the response 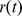 (Fig. 3 A 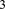) is simply a low-pass filtered version of the external input 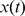 (Fig. 3 A 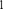), resulting in an exponentially decaying response auto-correlation (Fig. 3 D; light gray curve) and a drop in the response power-spectrum at the cutoff frequency 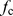 (Fig. 3 E). At low frequencies, 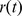 and 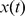 are in phase; they are correlated. In the presence of negative feedback (Fig. 3 B), the local input 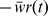 (Fig. 3 B 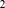) and the low-frequency components of the external input 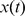 (Fig. 3 B 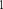) are anticorrelated. They partly cancel out, thereby reducing the response fluctuations 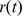 (Fig. 3 B 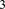). The auto-correlation function and the power-spectrum are suppressed (Fig. 3 D,E; black curves). Due to the low-pass characteristics of the system, mainly the low-frequency components of the external drive 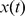 are transferred to the output side and, in turn, become available for the feedback signal. Therefore, the canceling of input fluctuations and the resulting suppression of response fluctuations are most efficient at low frequencies. Consequently, the auto-correlation function is sharpened (see inset in Fig. 3 D). The cutoff frequency of the system is increased (Fig. 3 E; black curve). This effect of negative feedback is very general and well known in the engineering literature. It is employed in the design of technical devices, like, e.g., amplifiers [47]. As the zero-frequency power is identical to the integrated auto-correlation function, the suppression of low-frequency fluctuations is accompanied by a reduction in the auto-correlation area (Fig. 3 D; black curve). Note that the suppression of fluctuations in the feedback case is not merely a result of the additional inhibitory noise source provided by the local input, but follows from the precise temporal alignment of the local and the external input. To illustrate this, let's consider the case where the feedback channel is replaced by a feedforward input 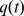 (Fig. 3 C) which has the same auto-statistics as the response 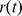 in the feedback case (Fig. 3 B 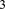) but is uncorrelated to the external drive 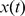. In this case, external input fluctuations (Fig. 3 C 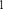) are not canceled by the local input 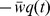 (Fig. 3 C 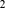). Instead, the local feedforward input acts as an additional noise source which leads to an increase in the response fluctuations (Fig. 3 C 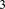). The response auto-correlation and power-spectrum (Fig. 3 D,E; dark gray curves) are increased. Compared to the unconnected case (Fig. 3 E; light gray curve), the cutoff frequency remains unchanged.

**Figure 4:**
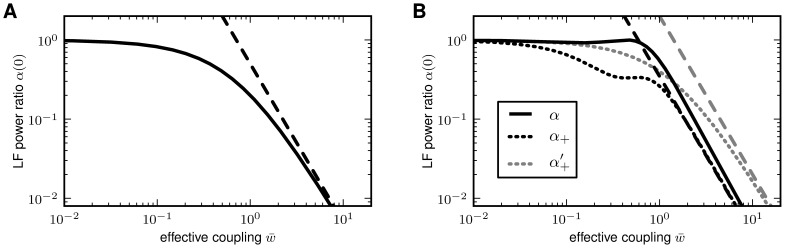
Suppression of low-frequency (LF) population-rate fluctuations in linearized homogeneous random networks with purely inhibitory (A) and mixed excitatory-inhibitory coupling (B). Dependence of the zero-frequency power ratio 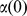 on the effective coupling strength 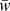 (solid curves: full solutions; dashed lines: strong-coupling approximations). The power ratio 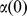 represents the ratio between the low-frequency population-rate power in the recurrent networks (**A**: Fig. 3 B; **B**: Fig. 5 A,B) and in networks where the feedback channels are replaced by uncorrelated feedforward input (**A**: Fig. 3 C; **B**, black: Fig. 5 C,D; **B**, gray: Fig. 5 D′). Dotted curves in **B** depict power ratio of the sum modes 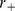 and 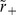 (see text). B: Balance factor 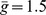.

The feedback induced suppression of response fluctuations can be quantified by comparing the response power-spectra


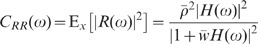
(3)

and


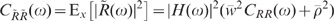
(4)

in the feedback (Fig. 3 B) and the feedforward case (Fig. 3 C), respectively (see **“** **Methods** **: Population-activity spectrum of the linear inhibitory network”**). Here, 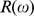 and 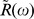 denote the Fourier transforms of the response fluctuations in the feedback and the feedforward scenario, respectively, 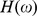 the transfer function (Fourier transform of the filter kernel 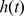) of the neuron population, and 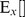 the average across noise realizations. We use the power ratio


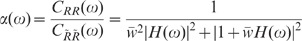
(5)

as a measure of the relative fluctuation suppression caused by feedback. For low frequencies (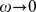) and strong effective coupling 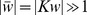, the power ratio (5) decays as 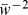 (see Fig. 4 A): the suppression of population-rate fluctuations is promoted by strong negative feedback. In line with the observations in **“** **Results** **: Suppression of population-rate fluctuations in LIF networks”**, this suppression is restricted to low frequencies; for high frequencies (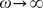, i.e. 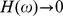), the power ratio 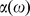 approaches 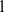. Note that the power ratio (5) is independent of the amplitude 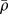 of the population averaged external input 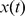. Therefore, even if we dropped the assumption of the external inputs 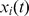 being uncorrelated, i.e. if 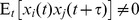 for 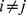, the power ratio (5) remained the same. For correlated external input, the power 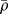 of the population average 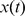 is different from 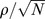. The suppression factor 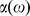, however, is not affected by this. Moreover, it is straightforward to show that the power ratio (5) is, in fact, independent of the shape of the external-noise spectrum 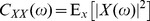. The same result (5) is obtained for any type of external input (e.g. colored noise or oscillating inputs).

For low frequencies, the transfer function 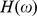 approaches unity (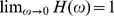); the exact shape of the kernel 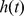 becomes irrelevant. In particular, the cutoff frequency (or time constant) of a low-pass kernel has no effect on the zero-frequency power (integral correlation) and the zero-frequency power ratio 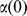 (Fig. 4). Therefore, the suppression of low-frequency fluctuations does not critically depend on the exact choice of the neuron, synapse or input model. The same reasoning applies to synaptic delays: Replacing the kernel 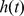 by a delayed kernel 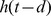 leads to an additional phase factor 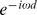 in the transfer function 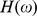. For sufficiently small frequencies (long time scales), this factor can be neglected (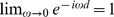).

For networks with purely inhibitory feedback, the absolute power (3) of the population rate decreases monotonously with increasing coupling strength 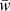. As we will demonstrate in **“** **Results** **: Population-activity fluctuations in excitatory-inhibitory networks”** and **“** **Results** **: Population averaged correlations in cortical networks”**, this is qualitatively different in networks with mixed excitatory and inhibitory coupling 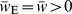 and 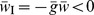, respectively: here, the fluctuations of the compound activity increase with 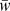. The power ratio 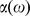, however, still decreases with 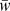.

### Population-activity fluctuations in excitatory-inhibitory networks

In the foregoing subsection, we have shown that negative feedback alone can efficiently suppress population-rate fluctuations and, hence, spike-train correlations. So far, it is unclear whether the same reasoning applies to networks with mixed excitatory and inhibitory coupling. To clarify this, we now consider a random network composed of a homogeneous excitatory and inhibitory subpopulation 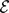 and 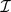 of size 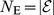 and 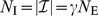, respectively. Each neuron receives 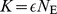 excitatory and 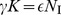 inhibitory inputs from 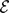 and 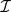 with synaptic weights 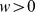 and 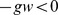, respectively. In addition, the neurons are driven by external Gaussian white noise. As demonstrated in **“** **Methods** **: Linearized network model”**, linearization and averaging across subpopulations leads to a two-dimensional system


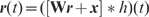
(6)

describing the linearized dynamics of the subpopulation averaged activity 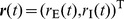. Here, 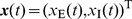 denotes the subpopulation averaged external uncorrelated white-noise input with correlation functions 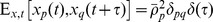 (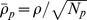, 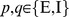), and 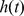 a normalized linear kernel with 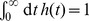. The excitatory and inhibitory subpopulations are coupled through an effective connectivity matrix


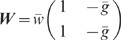
(7)

with effective weight 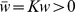 and balance parameter 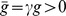.

**Figure 5:**
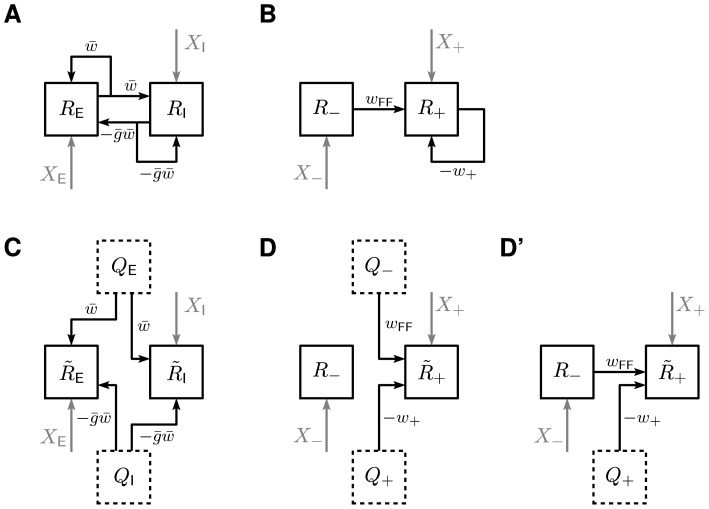
Sketch of the 2D (excitatory-inhibitory) model for the feedback (A,B) and the feedforward scenario (C,D) in normal (A,C) and Schur-basis representation (B,D). **A**: Original 2D recurrent system. **B**: Schur-basis representation of the system shown in A. **C**: Feedforward scenario: Excitatory and inhibitory feedback connections of the original network (A) are replaced by feedforward input from populations with rates 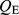, 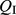, respectively. **D**: Schur-basis representation of the system shown in C. **D′**: Alternative feedforward scenario: Here, the feedforward channel (weight 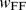) of the original system in Schur basis (B) remains intact. Only the inhibitory feedback (weight 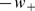) is replaced by feedforward input 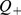.

The two-dimensional system (6)/(7) represents a recurrent system with both positive and negative feedback connections (Fig. 5 A). By introducing new coordinates


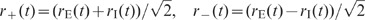
(8)

and 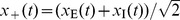, 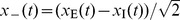, we obtain an equivalent representation of (6)/(7),


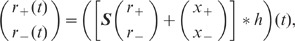
(9)

describing the dynamics of the sum and difference activity 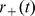 and 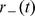, respectively, i.e. the in- and anti-phase components of the excitatory and inhibitory subpopulations (see [48]–[50]). The new coupling matrix


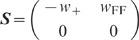
(10)

reveals that the sum mode 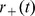 is subject to self-feedback (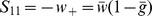) and receives feedforward input from the difference mode 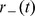 (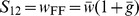). All remaining connections are absent (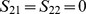) in the new representation (8) (see Fig. 5 B). The correlation functions of the external noise in the new coordinates are given by 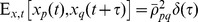 with 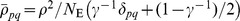 (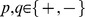).

The feedforward coupling is positive (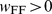): an excitation surplus (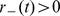) will excite all neurons in the network, an excitation deficit (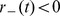) will lead to global inhibition. In inhibition dominated regimes with 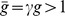, the self-feedback of the sum activity 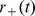 is effectively negative (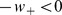). The dynamics of the sum rate in inhibition-dominated excitatory-inhibitory networks is therefore qualitatively similar to the dynamics in purely inhibitory networks (**“** **Results** **: Suppression of population-activity fluctuations by negative feedback”**). As shown below, the negative feedback loop exposed by the transform (8) leads to an efficient relative suppression of population-rate fluctuations (if compared to the feedforward case).

Mathematically, the coordinate transform (8) corresponds to a *Schur decomposition* of the dynamics: Any recurrent system of type (6) (with arbitrary coupling matrix 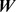) can be transformed to a system with a triangular coupling matrix (see, e.g., [50]). The resulting coupling between the different Schur modes can be ordered so that there are only connections from modes with lower index to modes with the same or larger index. In this sense, the resulting system has been termed ‘feedforward’ [50]. The original coupling matrix 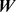 is typically not normal, i.e. 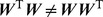. Its eigenvectors do not form an orthogonal basis. By performing a Gram-Schmidt orthonormalization of the eigenvectors, however, one can obtain a (normalized) orthogonal basis, a Schur basis. Our new coordinates (8) correspond to the amplitudes (the time evolution) of two orthogonal Schur modes.

The spectra 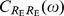, 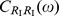, 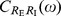 and 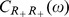 of the subpopulation averaged rates 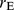, 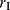 and the sum mode 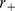, respectively, are derived in **“** **Methods** **: Population-activity spectra of the linear excitatory-inhibitory network”**. In contrast to the purely inhibitory network (see **“** **Results** **: Suppression of population-activity fluctuations by negative feedback”**), the population-rate fluctuations of the excitatory-inhibitory network increase monotonously with increasing coupling strength 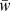. For strong coupling, 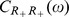 approaches


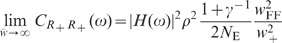
(11)

from below with 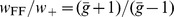. Close to the critical point (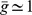), the rate fluctuations become very large; (11) diverges. Increasing the amount of inhibition by increasing 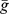, however, leads to a suppression of these fluctuations. In the limit 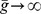, 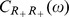 and (11) approach the spectrum 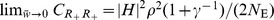 of the unconnected network. For strong coupling (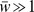), the ratio 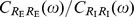 approaches 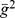: the fluctuations of the population averaged excitatory firing rate exceed those of the inhibitory population by a factor 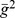 (independently of 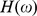 and 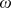).

Similarly to the strategy we followed in the previous subsections, we will now compare the population-rate fluctuations of the feedback system (6), or equivalently (9), to the case where the feedback channels are replaced by feedforward input with identical auto-statistics. A straight-forward implementation of this is illustrated in Fig. 5 C: Here, the excitatory and inhibitory feedback channels 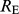 and 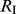 are replaced by uncorrelated feedforward inputs 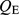 and 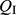, respectively. The Schur representation of this scenario is depicted in Fig. 5 D. According to (6), the Fourier transforms of the response fluctuations of this system read


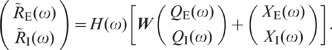
(12)

With 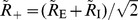, and using 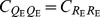, 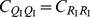, 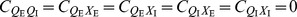, we can express the spectrum 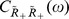 of the sum activity in the feedforward case in terms of the spectra 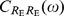 and 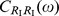 of the feedback system (see eq. (55)). For strong coupling (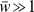), the zero-frequency component (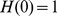) becomes


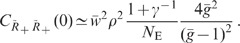
(13)

Thus, for strong coupling, the zero-frequency power ratio


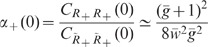
(14)

reveals a relative suppression of the population-rate fluctuations in the feedback system which is proportional to 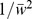 (see Fig. 4 B; black dashed line). The power ratio 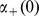 for arbitrary weights 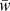 is depicted in Fig. 4 B (black dotted curve). For a network at the transition point 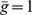, (14) equals 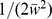. Increasing the level of inhibition by increasing 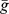 leads to a decrease in the power ratio: in the limit 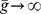, (14) approaches 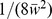 monotonously.

Above, we suggested that the negative self-feedback of the sum mode 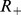, weighted by 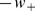 (Fig. 5 B), is responsible for the fluctuation suppression in the recurrent excitatory-inhibitory system. Here, we test this by considering the case where this feedback loop is opened and replaced by uncorrelated feedforward input 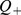, weighted by 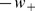, while the feedforward input from the difference mode 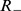, weighted by 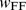, is left intact (see Fig. 5 D′). As before, we assume that the auto-statistics of 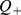 is identical to the auto-statistics of 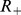 as obtained in the feedback case, i.e. 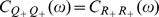. According to the Schur representation of the population dynamics (9)/(10), the Fourier transform of the sum mode of this modified system is given by


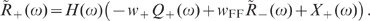
(15)

With 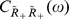 given in (54) and 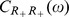, we obtain the power ratio


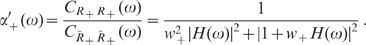
(16)

Its zero-frequency component 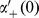 is shown in Fig. 4 B (gray dotted curve). For strong coupling, the power ratio decays as 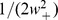 (gray dashed line in Fig. 4 B). Thus, the (relative) power in the recurrent system is reduced by strengthening the negative self-feedback loop, i.e. by increasing 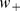.

So far, we have presented results for the subpopulation averaged firing rates 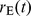 and 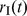 and the sum mode 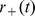. The spectrum of the compound rate 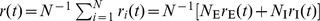, i.e. the activity averaged across the entire population, reads


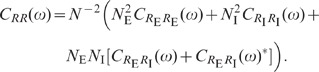
(17)

In the feedforward scenario depicted in Fig. 5 C, the spectrum of the compound rate 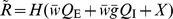 (with 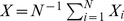) is given by


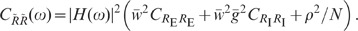
(18)

For strong coupling, the corresponding low-frequency power ratio 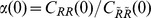 (black solid curve in Fig. 4 B) exhibits qualitatively the same decrease 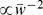 as the sum mode.

To summarize the results of this subsection: the population dynamics of a recurrent network with mixed excitatory and inhibitory coupling can be mapped to a two-dimensional system describing the dynamics of the sum and the difference of the excitatory and inhibitory subpopulation activities. This equivalent representation uncovers that, in inhibition dominated networks (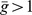), the sum activity is subject to negative self-feedback. Thus, the dynamics of the sum activity in excitatory-inhibitory networks is qualitatively similar to the population dynamics of purely inhibitory networks (see **“** **Results** **: Suppression of population-activity fluctuations by negative feedback”**). Indeed, the comparison of the compound power-spectra of the intact recurrent network and networks where the feedback channels are replaced by feedforward input reveals that the (effective) negative feedback in excitatory-inhibitory networks leads to an efficient suppression of population-rate fluctuations.

### Population averaged correlations in cortical networks

The results presented in the previous subsections describe the fluctuations of the compound activity. Pairwise correlations 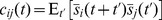 between the (centralized) spike trains 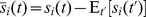 are outside the scope of such a description. In this subsection, we consider the same excitatory-inhibitory network as in **“** **Results** **: Population-activity fluctuations in excitatory-inhibitory networks”** and present a theory for the population averaged spike-train cross-correlations. In general, this is a hard problem. To understand the structure of cross-correlations, it is however sufficient to derive a relationship between the cross- and auto-covariances in the network, because the latter can, to good approximation, be understood in mean-field theory. The integral of the auto-covariance function of spiking LIF neurons can be calculated by Fokker-Planck formalism [12], [31], [51]. To determine the relation between the cross-covariance and the auto-covariance, we replace the spiking dynamics by a reduced linear model with covariances obeying, to linear order, the same relation. We present the full derivation in **“** **Methods** **: Linearized network model”**. There, we first derive an approximate linear relation between the auto- and cross-covariance functions 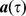 and 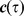, respectively, of the LIF network. A direct solution of this equation is difficult. In the second step, we therefore show that there exists a linear stochastic system with activity 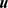 and correlations 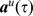 and 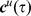 fulfilling the same equation as the original LIF model. This reduced model can be solved in the frequency domain by standard Fourier methods. Its solution allows us, by construction, to determine the relation between the integral cross-covariances 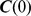 and the integral auto-covariances 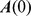 up to linear order.

**Figure 6:**
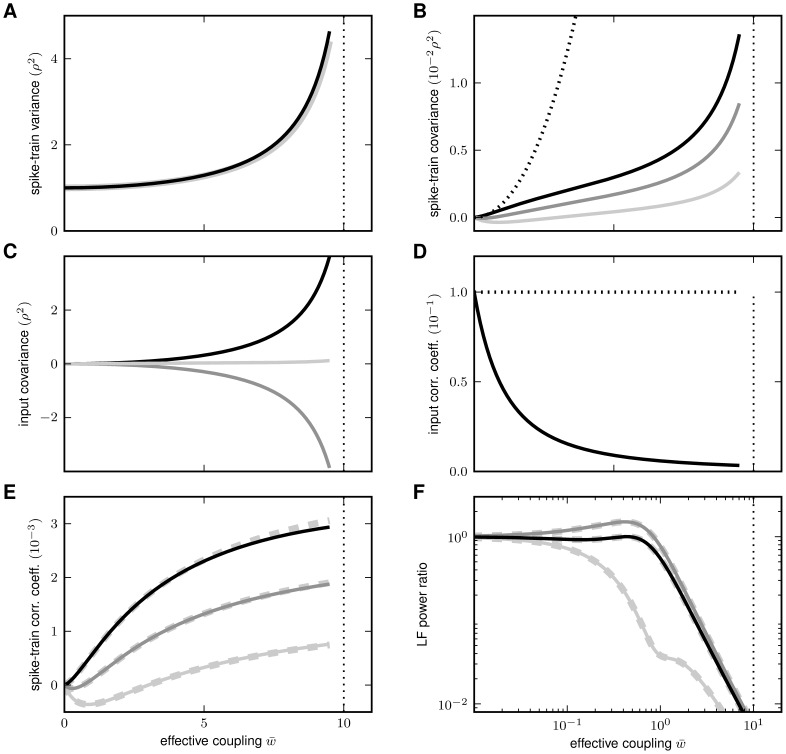
Dependence of population averaged correlations and population-rate fluctuations on the effective coupling 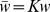 in a linearized homogeneous network with excitatory-inhibitory coupling. **A**: Spike-train variances 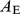 (black) and 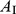 (gray) of excitatory and inhibitory neurons. **B**: Spike-train covariances 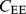 (black solid), 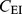 (dark gray solid) and 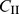 (light gray solid) for excitatory-excitatory, excitatory-inhibitory and inhibitory-inhibitory neuron pairs in the recurrent network, respectively, and shared-input contribution 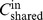 (black dotted curve; ‘feedforward case’). **C**: Decomposition of the total input covariance 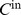 (light gray) into shared-input covariance 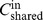 (black) and weighted spike-train covariance 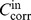 (dark gray). Covariances in A, B and C are given in units of the noise variance 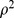. **D**: Input-correlation coefficient 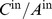 in the recurrent network (black solid curve). In the feedforward case, the input-correlation coefficient is identical to the network connectivity 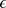 (horizontal dotted line). **E**: Spike-train correlation coefficients 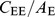 (black), 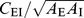 (dark gray) and 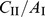 (solid light gray curve) for excitatory-excitatory, excitatory-inhibitory and inhibitory-inhibitory neuron pairs, respectively. Thick dashed curves represent approximate solutions assuming 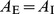. **F**: Low-frequency (LF) power ratios 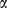 (black), 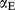 (dark gray), 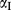 (solid light gray) for the population rate 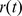 and the excitatory and inhibitory subpopulation rates 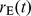 and 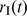, respectively. The LF power ratio represents the ratio between the LF spectra in the recurrent network and for the case where the feedback channels are replaced by feedforward input with 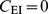 (cf. Fig. 5 C). Thick dashed curves in F show power ratios obtained by assuming that the auto-correlations are identical in the feedback and the feedforward scenario (see main text). Vertical dotted lines mark the stability limit of the linear model (see **“** **Methods** **: Linearized network model”**). A–F: 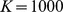, 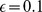, 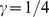, 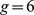, 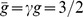, 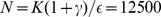.

As we are interested in the covariances averaged over many pairs of neurons, we average the resulting set of 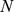 linear self-consistency equations (56) for the covariance matrix in the frequency domain 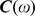 over statistically identical pairs of neurons and many realizations of the random connectivity (see **“** **Methods** **: Population averaged correlations in the linear EI network”**). This yields a four-dimensional linear system (76) describing the population averaged variances 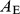 and 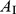 of the excitatory and inhibitory subpopulations, and the covariances 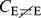 and 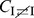 for unconnected excitatory-excitatory and inhibitory-inhibitory neuron pairs, respectively (note that we use the terms “variance” and “covariance” to describe the *integral* of the auto- and cross-correlation function, respectively; in many other studies, they refer to the zero-lag correlation functions instead). The dependence of the variances and covariances on the coupling strength 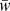, obtained by numerically solving (76), is shown in Fig. 6. We observe that the variances 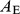 and 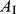 of excitatory and inhibitory neurons are barely distinguishable (Fig. 6 A). With the approximation 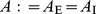, explicit expressions can be obtained for the covariances (thick dashed curves Fig. 6 E):


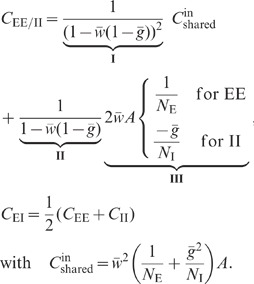
(19)

The deviations from the full solutions (thin solid curves in Fig. 6 E), i.e. for 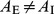, are small. In the reduced model, both the external input and the spiking of individual neurons contribute to an effective noise. As the fluctuations in the reduced model depend linearly on the amplitude 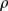 of this noise, the variances 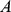 and covariances 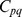 (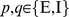) can be expressed in units of the noise variance 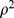. Consequently, the correlation coefficients 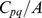 are independent of 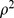 (see Fig. 6).

The analytical form (19) of the result shows that the correlations are smaller than expected given the amount of shared input a pair of neurons receives: The quantity 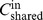 in the first line is the contribution of shared input to the covariance. For strong coupling 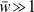, the prefactor 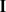 causes a suppression of this contribution. Its structure is typical for a feedback system, similar to the solution (3) of the one-population or the solution (52) of the two-population model. The term 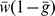 in the denominator represents the negative feedback of the compound rate. The prefactor 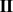 in the second line of (19) is again due to the feedback and suppresses the contribution of the factor 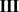, which represents the effect of direct connections between neurons.

Our results are consistent with a previous study of the decorrelation mechanism: In [17], the authors considered how correlations scale with the size 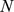 of the network where the synaptic weights are chosen as 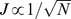. As a result, the covariance 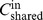 in (19) caused by shared input is independent of the network size, while the feedback 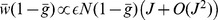 scales—to leading order—as 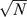 (see (45)). Consequently, the first line in (19) scales as 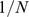. The same scaling holds for the second line in (19), explaining the decay of correlations as 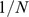 found in [17].

The first line in (19) is identical for any pair of neurons. The second line is positive for a pair of excitatory neurons and negative for a pair of inhibitory neurons. In other words, excitatory neurons are more correlated than inhibitory ones. Together with the third line in (19), this reveals a characteristic correlation structure: 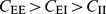 (Fig. 6 B,E). For strong coupling 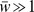, the difference between the excitatory and inhibitory covariance is 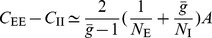. The difference decreases as the level 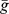 of inhibition is increased, i.e. the further the network is in the inhibition dominated regime, away from the critical point 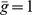.

To understand the suppression of shared-input correlations in recurrent excitatory-inhibitory networks, consider the correlation between the local inputs 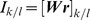 of a pair of neurons 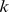, 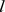. The input-correlation coefficient 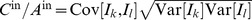 can be expressed in terms of the averaged spike-train covariances:


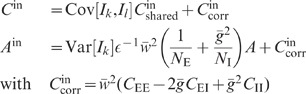
(20)

(see **“** **Methods** **: Population averaged correlations in the linear EI network”**: The input covariance 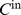 equals the average quantity 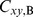 given in (67), the input variance 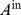 is given by (63) as 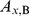). The term 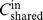 represents the contribution due to the spike-train variances of the shared presynaptic neurons (see (19)). This contribution is always positive (provided the network architecture is consistent with Dale's law; see [15]). In a purely feedforward scenario with uncorrelated presynaptic sources, 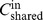 is the only contribution to the input covariance of postsynaptic neurons. The resulting response correlation for this feedforward case is much larger than in the feedback system (Fig. 6 B, black dotted curve). The correlation coefficient between inputs to a pair of neurons in the feedforward case is identical to the network connectivity 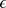 (horizontal dotted curve in Fig. 6 D; see [15]). In an inhibition dominated recurrent network, spike-train correlations between pairs of different source neurons contribute the additional term 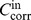, which is negative and of similar absolute value as the shared-input contribution 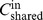. Thus, the two terms 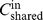 and 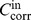 partly cancel each other (see Fig. 6 C). In consequence, the resulting input correlation coefficient 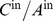 is smaller than 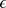 (see Fig. 6 D; here: 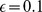).

The correlations in a purely inhibitory network can be obtained from (19) by replacing 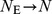, taking into account the negative sign of 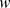 in 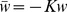 and setting 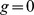 and 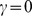:


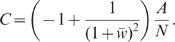
(21)

For finite coupling strength 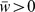, this expression is negative. The contributions of shared input and spike-train correlations to the input correlation are given by 
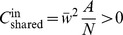
 and 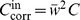, respectively (see (19) and (20)). Using (21), we can directly verify that 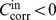, because pairwise correlations 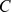 are negative, leading to a partial cancellation 
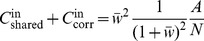
: the right hand side is smaller in magnitude by a factor of 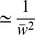 compared to each individual contribution. Hence, as in the network with excitation and inhibition, shared-input correlations are partly canceled by the contribution due to presynaptic pairwise spike-train correlations. In the feedforward scenario with zero presynaptic spike-train correlations, in contrast, the response correlations are determined by shared input alone and are therefore increased. The suppression of shared-input correlations in the feedback case is what we call ‘decorrelation’ in the current work. In purely inhibitory networks, this decorrelation is caused by weakly negative pairwise correlations (21). For sufficiently strong negative feedback, correlations are smaller in absolute value as compared to the feedforward case. The absolute value of these anti-correlations is bounded by 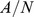.

The similarity in the results obtained for purely inhibitory networks and excitatory-inhibitory networks demonstrates that the suppression of pairwise correlations and population-activity fluctuations is a generic phenomenon in systems with negative feedback. It does not rely on an internal balance between excitation and inhibition.

As discussed in **“** **Results** **: Suppression of population-rate fluctuations in LIF networks”**, the suppression of correlations in the recurrent network is accompanied by a reduction of population-activity fluctuations. With the population averaged correlations (19), the power (1) of the population activity 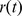 reads


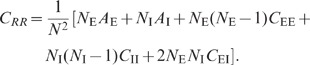
(22)

In **“** **Results** **: Population-activity fluctuations in excitatory-inhibitory networks”**, we showed that the population-activity fluctuations are amplified if the local input in the recurrent system is replaced by feedforward input from independent excitatory and inhibitory populations (see Fig. 5 C). This manipulation corresponds to a neglect of correlations 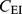 between excitatory and inhibitory neurons. All remaining correlations (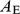, 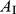, 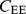, 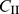) are preserved. With the resulting response auto- and cross-correlations 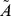 and 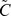 given by (84), the power (1) of the population activity becomes


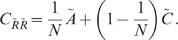
(23)

For large effective coupling 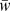, the power ratio 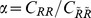 decays as 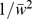 (black curve in Fig. 6 F). Note that the power ratio 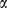 derived here is indistinguishable from the one we obtained in the framework of the population model in **“** **Results** **: Population-activity fluctuations in excitatory-inhibitory networks”** (black solid curve in Fig. 4 B). Although the derivation of the macroscopic model in **“** **Results** **: Population-activity fluctuations in excitatory-inhibitory networks”** is different from the one leading to the population averaged correlations described here, the two models are consistent: They describe one and the same system and lead to identical power ratios.

The fluctuation suppression is not only observed at the level of the entire network, i.e. for the population activity 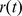, but also for each individual subpopulation 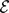 and 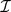, i.e. for the subpopulation averaged activities 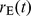 and 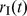. The derivation of the corresponding power ratios 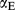 and 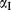 is analog to the one described above. As a result of the correlation structure 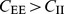 in the feedback system (see Fig. 6 B), the power of the inhibitory population activity is smaller than the power of the excitatory population activity. In consequence, 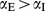 (gray curves in Fig. 6 F).

In (22) and (23), the auto-correlations are scaled by 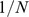, while the cross-correlations enter with a prefactor of order unity. For large 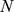, one may therefore expect that the suppression of population-activity fluctuations is essentially mediated by pairwise correlations. In the recurrent system, however, the cross-correlations 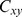 (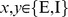) are of order 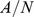 (see Fig. 6 and (19)). It is therefore a priori not clear whether the fluctuation suppression is indeed dominated by pairwise correlations. In our framework, one can explicitly show that the auto-correlation is irrelevant: Replacing the auto-correlation 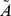 in (23) by the average auto-correlation 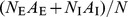 of the intact feedback system has no visible effect on the resulting power ratio (dashed curves in Fig. 6 F). The difference in the spectra of the population activities 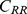 and 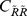 is therefore essentially caused by the cross-correlations.

The absolute population-activity fluctuations in purely inhibitory and in excitatory-inhibitory networks show a qualitatively different dependence on the synaptic coupling 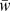, in agreement with the previous sections. In networks with excitation and inhibition, the correlation coefficient increases with increasing synaptic coupling (see Fig. 6 E). Hence, the population-activity fluctuations grow with increasing coupling strength. In purely inhibitory networks, in contrast, the pairwise spike-train correlation decreases monotonously with increasing magnitude of the coupling strength 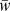, see (21). In consequence, the population-activity fluctuations decrease. The underlying reason is that, in the inhibitory network, the power of the population activity is directly proportional to the covariance of the input currents, which is actively suppressed, as shown above. For excitatory-inhibitory networks, these two quantities are not proportional (compare (20) and (1)) due to the different synaptic weights appearing in the input covariance.

**Figure 7:**
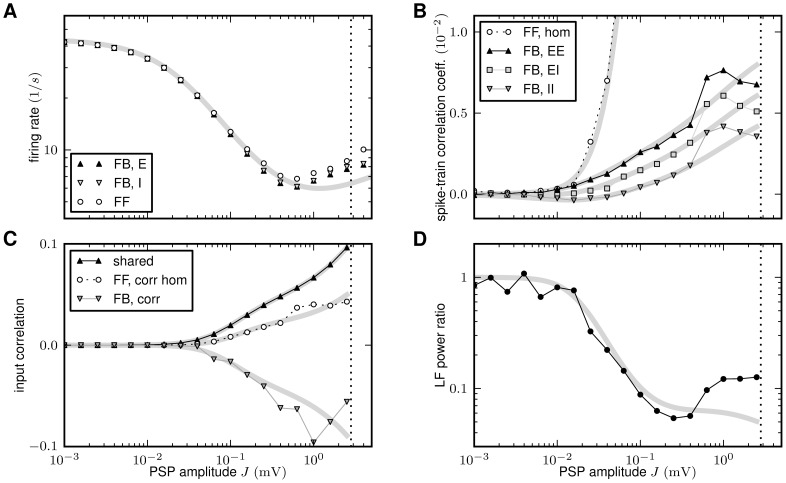
Comparison between predictions of the linear theory (thick gray curves) and direct simulation of the LIF-network model (symbols and thin lines). Dependence of the spike-train and population-rate statistics on the synaptic weight 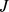 (PSP amplitude) in a recurrent excitatory-inhibitory network (‘feedback system’, ‘FB’) and in a population of unconnected neurons receiving randomized feedforward input (‘feedforward system’, ‘FF’) from neurons in the recurrent network. Average presynaptic firing rates and shared-input structure are identical in the two systems. In the FF case, the average correlations between presynaptic spike-trains are homogenized (i.e. 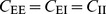) as a result of the random reassignment of presynaptic neuron types. The mapping of the LIF dynamics to the linear reduced dynamics (**“** **Methods** **: Response kernel of the LIF model”**) relates the PSP amplitude 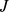 to the effective coupling strength 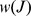 by (45), as shown in Fig. 8 B. **A**: Average firing rates 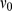 in the FB (black up-triangles: excitatory neurons; gray down-triangles: inhibitory neurons) and in the FF system (open circles). Analytical prediction (??) (gray curve). **B**: Spike-train correlation coefficients 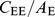 (black up-triangles), 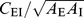 (gray squares) and 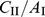 (gray down-triangles) for excitatory-excitatory, excitatory-inhibitory, and inhibitory-inhibitory neuron pairs, respectively, in the FB system. Analytical prediction (19) (gray curves). Spike-train correlation coefficient 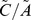 (open circles) in the FF system with homogenized presynaptic spike-train correlations. Analytical prediction (86) (underlying gray curve). **C**: Shared-input (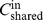; black up-triangles) and spike-correlation contribution 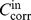 (FB: gray down-triangles; FF: open circles) to the input correlation 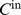 (normalized by 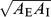). Analytical predictions (20). **D**: Low-frequency (LF) power ratio of the compound activity. Vertical dotted lines in A–D mark the stability limit of the linear model (see **“** **Methods** **: Linearized network model”**). 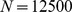, 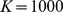, 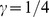, 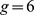. Size of postsynaptic population in the FF case: 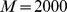. Simulation time: 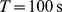.

**Figure 8:**
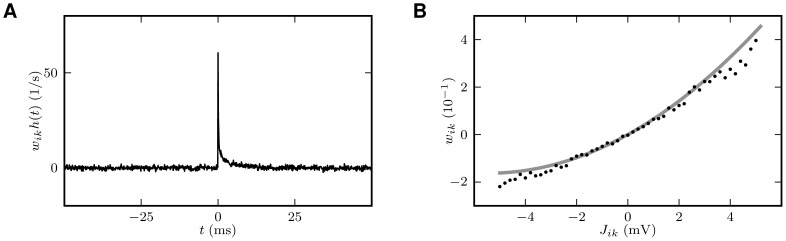
Linear response and relation between synaptic weight 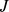 and effective coupling strength 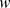. **A**: Firing-rate deflection 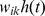 of a LIF neuron caused by an incoming spike event of postsynaptic amplitude 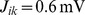. **B**: Integral 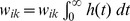 of the firing rate deflection shown in A as a function of the postsynaptic amplitude 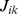 (simulation: black dots; analytical approximation (45) : gray curve). The neuron receives constant synaptic background input with 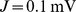, 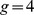, and rates 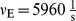, 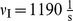 resulting in a first and second moment (42) 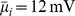 and 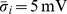. Simulation results are obtained by averaging over 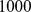 trials of 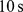 duration each with 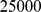 input impulses on average. For further parameters of the neuron model, see Table 1 and Table 2.

To compare our theory to simulations of spiking LIF networks, we need to determine the effect of a synaptic input on the response activity of the neuron model. To this end, we employ the Fokker-Planck theory of the LIF model (see **“** **Methods** **: Response kernel of the LIF model”**). In this context, the steady state of the recurrent network is characterized by the mean 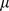 and the standard deviation 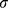 of the total synaptic input. Both 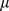 and 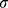 depend on the steady-state firing rate in the network. The steady-state firing rate can be determined in a self-consistent manner [12] as the fixed point of the firing rate approximation (42). The approximation predicts the firing rate to sufficient accuracy of about 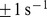 (see Fig. 7 A). We then obtain an analytical expression of the low-frequency transfer which relates the fluctuation 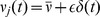 of a synaptic input to neuron 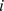 to the fluctuation of neuron 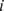
's response firing rate to linear order, so that 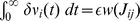. This relates the postsynaptic potential 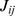 in the LIF model to the effective linear coupling 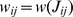 in our linear theory. The functional relation 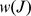 can be derived in analytical form by linearization of (42) about the steady-state working point. Note that 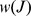 depends on 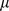 and 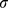 and, hence, on the steady-state firing rate in the network. The derivation outlined in **“** **Methods** **: Response kernel of the LIF model”** constitutes an extension of earlier work [21], [33] to quadratic order in 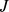. The results agree well with those obtained by direct simulation for a large range of synaptic amplitudes (see Fig. 8).

Fig. 7 B compares the population averaged correlation coefficients 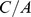 obtained from the linear reduced model, see (19), and simulations of LIF networks. Note that the absolute value of the noise amplitude 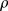 in the reduced model does not influence the correlation coefficient 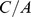, as both quantities 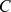 and 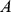 depend linearly on 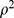. Theory and simulation agree well for synaptic weights up to 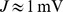. For larger synaptic amplitudes, the approximation of the effective linear transfer for a single neuron obtained from the Fokker-Planck theory deviates from its actual value (see Fig. 8 B). Fig. 7 C shows that the cancellation of the input covariance in the LIF network is well explained by the theory.

Previous work [17] suggested that positive correlations between excitatory and inhibitory inputs lead to a negative component in the input correlation which, in turn, suppresses shared-input correlations. The mere existence of positive correlations between excitatory and inhibitory inputs is however not sufficient. To explain the effect, it is necessary to take the particular correlation structure 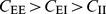 into account. To illustrate this, consider the case where the correlation structure is destroyed by replacing all pairwise correlations in the input spike-train ensemble by the overall population average 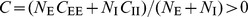 (homogenization of correlations). The resulting response correlations (upper gray curve in Fig. 7 B) are derived in **“** **Methods** **: Population averaged correlations in the linear EI network”**, eq. (86). In simulations of LIF networks, we study the effect of homogenized spike-train correlations by first recording the activity of the intact recurrent network, randomly reassigning the neuron type (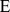 or 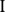) to each recorded spike train, and feeding this activity into a second population of neurons. Compared to the intact recurrent network, the response correlations are significantly larger (Fig. 7 B). The contribution of homogenized spike-train correlations to the input covariance 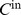 (see (20)) is given by 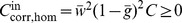. For positive spike-train correlations 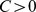, this contribution is greater or equal zero (zero for 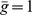). Hence, it cannot compensate the (positive) shared-input contribution 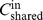 (see Fig. 7 C). In consequence, input correlations, output correlations and, in turn, population-rate fluctuations (Fig. 7 D) cannot be suppressed by homogeneous positive correlations in the input spike-train ensemble. Canceling of shared-input correlations requires either negative spike-train correlations (as in purely inhibitory networks) or a heterogeneity in correlations across different pairs of neurons (e.g. 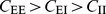).

### Effect of feedback manipulations

In the previous subsections, we quantified the suppression of population-rate fluctuations in recurrent networks by comparing the activity in the intact recurrent system (feedback scenario) to the case where the feedback is replaced by feedforward input with some predefined statistics (feedforward scenario). We particularly studied the effect of neglecting the auto-statistics of the compound feedback, (the structure of) correlations within the feedback ensemble and/or correlations between the feedback and the external input. In all cases, we observed a significant amplification of population-activity fluctuations in the feedforward scenario. In this subsection, we further investigate the role of different types of feedback manipulations by means of simulations of LIF networks with excitatory-inhibitory coupling. To this end, we record the spiking activity of the recurrent network (feedback case), apply different types of manipulations to this activity (described in detail below) and feed this modified activity into a second population of identical (unconnected) neurons (feedforward case). As before, the connectivity structure (in-degrees, shared-input structure, synaptic weights) is exactly identical in the feedback and the feedforward case.

**Figure 9:**
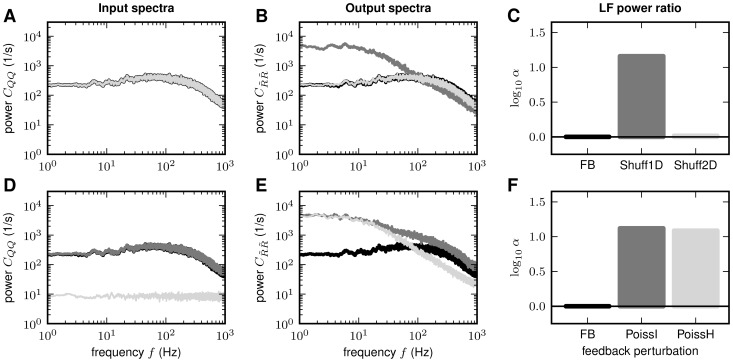
Amplification of population-rate fluctuations by different types of feedback manipulations in a random network of excitatory and inhibitory LIF neurons (simulation results). Top row (**A**–**C**): Unperturbed feedback (FB; black), shuffling of spike-train senders across entire network (Shuff1D; dark gray) and within each subpopulation (E,I) separately (Shuff2D; light gray). Bottom row (**D**–**F**): Unperturbed feedback (FB; black), replacement of spike trains by realizations of inhomogeneous (PoissI; dark gray) and homogeneous Poisson processes (PoissH; light gray). In the PoissI (PoissH) case, the (time averaged) subpopulation rates are approximately preserved. **A**, **D**: Compound power-spectra 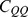 of input spike-train ensembles. **B**, **E**: Power-spectra 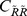 of population-response rates. **C**, **F**: Low-frequency (LF; 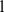
–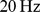) power ratio 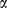 (increase in LF power relative to the unperturbed case [FB]; logarithmic scaling). Note that in A, the compound-input spectra (FB, Shuff1D, Shuff2D) are identical. In D, the input spectra for the intact recurrent network (FB) and the inhomogeneous-Poisson case (PoissI) are barely distinguishable. See Table 1 and Table 2 for details on the network model and parameters. Simulation time 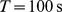. Single-trial spectra smoothed by moving average (frame size 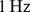).

In **“** **Methods** **: Linearized network model”**, we show that the low-frequency fluctuations of the population rate 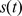 of the spiking model are captured by the reduced model 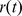 presented in the previous subsections. To verify that the theory based on excitatory and inhibitory population rates is indeed sufficient to explain the decorrelation mechanism, we first consider the case where the sender identities of the presynaptic spike train are randomly shuffled. Fig. 9 A shows the power-spectrum of the population activity recorded in the original network (FB) as well as the spectra obtained after shuffling spike-train identities within the excitatory and inhibitory subpopulations separately (Shuff2D), or across the entire network (Shuff1D). As shuffling of neuron identities does not change the population rates, all three compound spectra are identical. Fig. 9 B shows the response power-spectra of the neuron population receiving the shuffled spike trains. Shuffling within the subpopulations (Shuff2D) preserves the population-specific fluctuations and average correlations. The effect on the response fluctuations is negligible (compare black and light gray curves in Fig. 9 B). In particular, the power of low-frequency fluctuations remains unchanged (Fig. 9 C). This result confirms that population models which take excitatory and inhibitory activity separately into account are sufficient to explain the observations. Shuffling of spike-train identities across subpopulations (Shuff1D), in contrast, causes an increase in the population fluctuations by about one order of magnitude (Fig. 9 B,C; dark gray). This outcome is in agreement with the result obtained by homogenizing pairwise correlations (see Fig. 7) and demonstrates that the excitatory and inhibitory subpopulation rates have to be conserved to explain the observed fluctuation suppression.

The shuffling experiments and the results of the linear model in the previous subsections suggest that the precise temporal structure of the *population averaged* activities within homogeneous subpopulations is essential for the suppression of population-rate fluctuations. Preserving the exact structure of individual spike trains is not required. This is confirmed by simulation experiments where new sender identities were randomly reassigned for each individual presynaptic spike (rather than for each spike train; data not shown). This operation destroys the structure of individual spike trains but preserves the compound activities. The results are similar to those reported here.

So far, it is unclear how sensitive the fluctuation-suppression mechanism is to perturbations of the temporal structure of the population rates. To address this question, we replaced the excitatory and inhibitory spike trains in the feedback ensemble by independent realizations of inhomogeneous Poisson processes (PoissI) with intensities given by the measured excitatory and inhibitory population rates 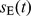 and 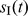 of the recurrent network, respectively. Note that the compound rates of a single realization of this new spike-train ensemble are similar but not identical to the original population rates 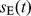, 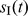 (in each time window 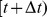, the resulting spike count is a random number drawn from a Poisson distribution with mean and variance proportional to 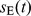 and 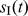, respectively). Although the compound spectrum of the resulting local input is barely distinguishable from the compound spectrum of the intact recurrent system (Fig. 9 D; black and dark gray curves), the response spectra are very different: replacing the feedback ensemble by inhomogeneous Poisson processes leads to a substantial amplification of low-frequency fluctuations (Fig. 9 E; compare black and dark gray curves). The effect is as strong as if the temporal structure of the population rates was completely ignored, i.e. if the feedback channels were replaced by realizations of homogeneous Poisson processes with constant rates (PoissH; light gray curves in Fig. 9 D,E). This result indicates that the precise temporal structure of the population rates is essential and that even small deviations can significantly weaken the fluctuation-suppression mechanism. The results of the Poisson experiments can be understood by considering the effect of the additional noise caused by the stochastic realization of individual spikes. Considering the auto-correlation, a Poisson spike-train ensemble with rate profile 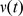 is equivalent to a sum of the rate profile and a noise term resulting from the stochastic (Poissonian) realization of spikes, 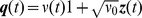. Here, 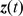 denotes a Gaussian white noise with auto-correlation 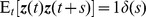 and 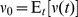 the mean firing rate. The response fluctuations of the population driven by the rate modulated Poisson activity are, to linear approximation, given by 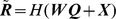. Inserting 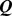, we obtain an additional noise term 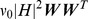 in the spectrum 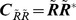 which explains the increase in power compared to the spectrum 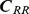 of the recurrent network. As a generalization of the Poisson model, one may replace the noise amplitude 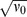 by some arbitrary prefactor 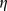. In simulation experiments, we observed a gradual amplification of the population-rate fluctuations with increasing noise amplitude 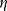 (data not shown).

## Discussion

We have shown that negative feedback in recurrent neural networks actively suppresses low-frequency fluctuations of the population activity and pairwise correlations. This mechanism allows neurons to fire more independently than expected given the amount of shared presynaptic input. We demonstrated that manipulations of the feedback statistics, e.g. replacing feedback by uncorrelated feedforward input, can lead to a significant amplification of response correlations and population-rate fluctuations.

The suppression of correlations and population-rate fluctuations by feedback can be observed in networks with both purely inhibitory and mixed excitatory-inhibitory coupling. In purely inhibitory networks, the effect can be understood by studying the role of the effective negative feedback experienced by the compound activity. In networks of excitatory and inhibitory neurons, a change of coordinates, technically a Schur decomposition, exposes the underlying feedback structure: the sum of the excitatory and inhibitory activity couples negatively to itself if the network is in an inhibition dominated regime (which is required for its stability; see, e.g., [12). This negative feedback suppresses fluctuations in a similar way as in purely inhibitory networks. The fluctuation suppression becomes more efficient the further the network is brought into the inhibition dominated regime, away from the critical point of equal recurrent excitation and inhibition (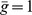). Having identified negative feedback as the underlying cause of small fluctuations and correlations, we can rule out previous explanations based on a balance between (correlated) excitation and inhibition [17]. We presented a self-consistent theory for the average pairwise spike-train correlations which illuminates that the suppression of population-rate fluctuations and the suppression of pairwise correlations are two expressions of the same effect: as the single spike-train auto-covariance is the same in the feedforward and the feedback case, the suppression of population-rate fluctuations implies smaller correlations. Our theory enables us to identify the cancellation of input correlations as a hallmark of small spike-train correlations.

In previous studies, shared presynaptic input has often been considered a main source of correlation in recurrent networks (e.g. [15], [52]). Recently [17], suspected that correlations between excitatory and inhibitory neurons and the fast tracking of external input by the excitatory and the inhibitory population are responsible for an active decorrelation. We have demonstrated here that the mere fact that excitatory and inhibitory neurons are correlated is not sufficient to suppress shared-input correlations. Rather, we find that the spike-train correlation structure in networks of excitatory and inhibitory networks arranges such that their overall contribution to the covariance between the summed inputs to a pair of neurons becomes negative, canceling partly the effect of shared inputs. This cancellation becomes more precise the stronger the negative compound feedback 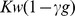 is. In homogeneous networks where excitatory and inhibitory neurons receive statistically identical input, the particular structure of correlations is 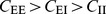. It can further be shown that this structure of correlations is preserved in the limit of large networks 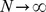 (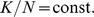). For non-homogeneous synaptic connectivity, if the synaptic amplitudes depend on the type of the target neuron (i.e. 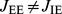 or 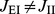), the structure of correlations may be different. Still, the correlation structure arranges such that shared input correlation is effectively suppressed. Formally, this can be seen from a self-consistency equation similar to our equation (80).

The study by [17] has shown that correlations are suppressed in the limit of infinitely large networks of binary neurons receiving randomly drawn inputs from a common external population. Its argument rests on the insight that the population-activity fluctuations in a recurrent balanced network follow the fluctuations of the external common population. An elegant scaling consideration for infinitely large networks 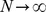 with vanishing synaptic efficacy 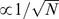 shows that this fast tracking becomes perfect in the limit. This allows to determine the zero-lag pairwise correlations caused by the external input. The analysis methods and the recurrent networks presented here differ in several respects from these previous results: We study networks of a finite number of spiking model neurons. The neurons receive uncorrelated external input, so that correlations are due to the local recurrent connectivity among neurons, not due to tracking of the common external input [17]. Moreover, we consider homogeneous connectivity where synaptic weights depend only on the type of the presynaptic neuron (as, e.g., in [12]), resulting in a correlation structure 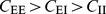. For such connectivity, networks of binary neurons with uncorrelated external input exhibit qualitatively the same correlation structure as reported here (results not shown).

In purely inhibitory networks, the decorrelation occurs in an analog manner as in excitatory-inhibitory networks. As only a single population of neurons is available here, population averaged spike-train correlations 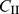 are negative. This negative contribution compensates the positive contribution of shared input.

The structure of integrated spike-train covariances in networks constitutes an experimentally testable prediction. Note, however, that the prediction (19) obtained in the current work rests on two simplifying assumptions: identical internal dynamics of excitatory and inhibitory neurons and homogeneous connectivity (i.e. 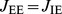, 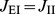; see **“** **Results** **: Population-activity fluctuations in excitatory-inhibitory networks”**). For such networks, the structure of correlations is given by 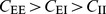. Further, the relation between subthreshold membrane-potential fluctuations and spike responses is the same for both neuron types. Consequently, the above correlation structure can be observed not only at the level of spike trains but also for membrane potentials, provided the assumptions hold true. A recent experimental study [53] reports neuron-type specific cross-correlation functions in the barrel cortex of behaving mice, both for spike trains and membrane potentials. It is however difficult to assess the integral correlations from the published data. A direct test of our predictions requires either a reanalysis of the data or a theory predicting the entire correlation functions. The raw (unnormalized) II and EI spike-train correlations in [53] are much more pronounced than the EE correlations (Fig. 6 in [53]). This seems to be in contradiction to our results. Note, however, that the firing rates of excitatory and inhibitory neurons are very different in [53]. In our study, in contrast, the average firing rates of excitatory and inhibitory neurons are identical as a consequence of the assumed network homogeneity. Future theoretical work is needed to generalize our model to networks with heterogeneous firing rates and non-homogeneous connectivity. Recent results on the dependence of the correlation structure on the connectivity may prove useful in this endeavor [54]–[56].

Correlations in spike-train ensembles play a crucial role for the en- and decoding of information. A set of uncorrelated spike trains provides a rich dynamical basis which allows readout neurons to generate a variety of responses by tuning the strength and filter properties of their synapses [1]

In the presence of correlations, the number of possible readout signals is limited. Moreover, spike-train correlations impair the precision of such readout signals in the presence of noise. Consider, for example, a linear combination 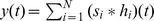 of 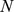 presynaptic spike trains with arbitrary (linear) filter kernels 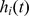 (e.g. synaptic filters). In a realistic scenario, the individual spike trains 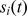 typically vary across trials [3], [57]. To understand how robust the resulting readout signal 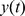 is against this spike-train variability, let's consider the variability of its Fourier transform 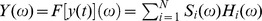. Assuming homogeneous spike-train statistics,


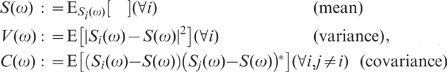
(24)

the (squared) signal-to-noise ratio of the readout signal 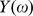 is given by


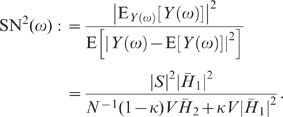
(25)

Here, 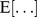 denotes the average across the ensemble of spike-train realizations, 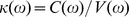 the spike-train coherence, and the coefficients 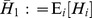 and 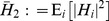 the 1st- and 2nd-order filter statistics. For uncorrelated spike trains, i.e. 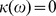, and 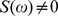, the signal-to-noise ratio 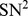 grows unbounded with the population size 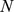. Thus, even for noisy spike trains (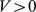), the compound signal 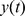 can be highly reliable if the population size 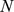 is sufficiently large. In the presence of correlations, 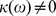, however, 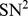 converges towards a constant value 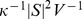 as 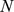 grows. Even for large populations, the readout signal remains prone to noise. These findings constitute a generalization of the results reported for population-rate coding, i.e. sums of unweighted spike counts (see, e.g., [2], [3]). The above arguments illustrate that the same reasoning applies to coding schemes which are based on the spatio-temporal structure of spike patterns.

**Figure 10:**
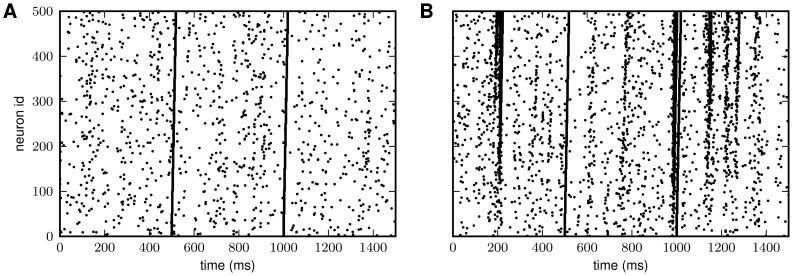
Recurrent network dynamics stabilizes dynamics of embedded synfire chains. Spiking activity in a synfire chain (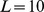 layers, layer width 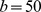) receiving background input from an excitatory-inhibitory network (**A**, cf. Fig. 1 C) or from a finite pool of excitatory and inhibitory Poisson processes (**B**, cf. Fig. 1 D). Average input firing rates, in-degrees and amount of shared input are identical in both cases. Neurons of the first synfire layer (neuron ids 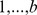) are stimulated by current pulses at times 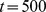 and 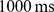. Each neuron in layer 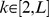 receives inputs from all 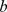 neurons in the preceding layer 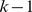 (synaptic weights 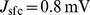, spike transmission delays 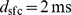), and 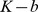 and 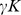 excitatory and inhibitory background inputs, respectively, randomly drawn from the presynaptic populations. Neurons in the first layer 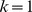 receive 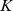 and 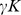 excitatory and inhibitory background inputs, respectively. Note that there is no feedback from the synfire chain to the embedding network. See Table 2 for network parameters.

In a previous study [9], we demonstrated that active decorrelation in recurrent networks is a necessary prerequisite for a controlled propagation of synchronous volleys of spikes in embedded feedforward subnetworks (‘synfire chains’; Fig. 10): A synfire chain receiving background input from a finite population of independent Poisson sources amplifies the resulting shared-input correlations, thereby leading to spontaneous synchronization within the chain (Fig. 10 B). A distinction between these spurious synchronous events and those triggered by an external stimulus is impossible. The synfire chain loses its asynchronous ground state [58]. A synfire chain receiving background inputs from a recurrent network, in contrast, is much more robust. Here, shared-input correlations are actively suppressed by the recurrent-network dynamics. Synchronous events can be triggered by external stimuli in a controlled manner (Fig. 10 A). Apart from the spontaneous synchronization illustrated in Fig. 10, decorrelation by inhibition might solve another problem arising in embedded synfire structures: In the presence of feedback connections between the synfire chain and the embedding background network, synchronous spike volleys can excite (high-frequency) oscillatory modes in the background network which, in turn, interfere with the synfire dynamics and prevent a robust propagation of synchronous activity within the chain (‘synfire explosion’; see [59], [60]). The decorrelation mechanism we refer to in our work is efficient only at low frequencies. It cannot prevent the build-up of these oscillations. [61] demonstrated that the ‘synfire explosion’ can be suppressed by adding inhibitory neurons to each synfire layer (‘shadow inhibition’) which diffusely project to neurons in the embedding network, thereby weakening the impact of synfire activity on the embedding network.

In the present work we focus on the integral of the correlation function, nurtured by our interest in the low-frequency fluctuations. An analog treatment can however easily be performed for the zero-lag correlations. In contrast to infinite networks with sparse connectivity (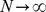, 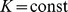), in the case of finite networks, pairs of neurons must be distinguished according to whether they are synaptically connected or not in order to arrive at a self-consistent theory for the averaged correlations. Providing explicit expressions for correlations between connected and unconnected neurons, the current work provides the tools to relate experimentally observed spiking correlations to the underlying synaptic connectivity.

The quantification of pairwise correlations is a necessary prerequisite to understand how correlation sensitive synaptic plasticity rules, like spike-timing dependent plasticity [62], interact with the recurrent network dynamics [63]. Existing theories quantifying correlations employ stochastic neuron models and are limited to purely excitatory networks [63]–[65]. Here, we provide an analytical equivalence relation between a reduced linear model and spiking integrate-and-fire neurons describing fluctuations correctly up to linear order. A formally similar approach has been employed earlier to study delayed cumulative inhibition in spiking networks [66]. We show that the correlations observed in recurrent networks in the asynchronous irregular regime are quantitatively captured for realistic synaptic coupling with postsynaptic potentials of up to about 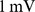. The success of this approach can be explained by the linearization of the neural threshold units by the afferent noise experienced in the asynchronous regime. For linear neural dynamics, the second-order description of fluctuations is closed [67]. We exploit this finding by applying perturbation theory to the Fokker-Planck description of the integrate-and-fire neuron to obtain the linear input-output transfer at low frequencies [33], thereby determining the effective coupling in our linear model.

The scope of the theory presented in the current work is limited mainly by three assumptions. The first is the use of a linear theory which exhibits an instability as soon as a single eigenvalue of the effective connectivity matrix assumes a positive real part. This ultimately happens when increasing the synaptic coupling strength, because the eigenvalues of the random connectivity matrix are located in a circle centered in the left half of the complex plain with a radius given by the square root of the variance of the matrix elements [68], [69]. Nonlinearities, like those imposed by strictly positive firing rates, prevent such unbounded growth (or decay) by saturation. For nonlinear rate models with sigmoidal transfer functions it has been shown that the activity of recurrent random networks of such units makes a transition to chaos at the point where the linearized dynamics would loose stability [70]. However, this point of transition is sharp only in the limit of infinitely large networks. From the population averaged firing rate and the pairwise correlations averaged over pairs of neurons considered in Fig. 7 we cannot conclude whether or not a transition to chaos occurs in the spiking network. In simulations and in the linearized reduced model, we could however observe that the distribution of pairwise correlations broadens when approaching the point of instability. Future work needs to examine this question in detail, e.g. by considering measures related to the Lyapunov exponent. Recently developed semi-analytical theories accounting for nonlinear neural features [71] may be helpful to answer this question. The second limiting factor of the current theory is the use of a perturbative approach to quantify the response of the integrate-and-fire model. Although the steady-state firing rate of the network is found as the fixed point of the nonlinear self-consistency equation, the response to a synaptic fluctuation is determined up to linear order in the amplitude of the afferent rate fluctuation, which is only valid for sufficiently small fluctuations. For larger input fluctuations, nonlinear contributions to the neural response can become more important [33]. Also for strong synaptic coupling, deviations from our theory are to be expected. Thirdly, the employment of Fokker-Planck theory to determine the steady-state firing rate and the response to incoming fluctuations assumes uncorrelated presynaptic firing with Poisson statistics and synaptic amplitudes which are vanishingly small compared to the distance between reset and threshold. For larger synaptic amplitudes, the Fokker-Planck theory becomes approximate and deviations are expected [33], [34], [72], [73]. This can be observed in Fig. 7 A, showing a deviation between the self-consistent firing rate and the analytical prediction at about 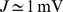. In this work, we obtained a sufficiently precise self-consistent approximation of the correlation coefficient 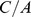 by relating the random recurrent network of spiking neurons in the asynchronous irregular state to a reduced linear model which obeys the same relation between 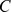 and 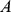 up to linear order. This reduced linear model, however, does not predict the absolute values of the variance 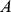 and covariance 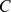. The variance 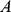 of the LIF model, for example, is dominated by nonlinear effects, such as the reset mechanism after each action potential. Previous work [12], [31] has shown that the single spike-train statistics can be approximated in the diffusion approximation if the recurrent firing rate in the network is determined by mean-field theory. One may therefore extend our approach and determine the integral auto-correlation function as 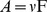 with the Fano factor 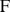 (see [51]). For a renewal process and long observation times, the Fano factor is given by 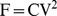 [74], [75]. The coefficient of variation 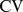 can be obtained from the diffusion approximation of the membrane-potential dynamics (App. A.1 in [12). The covariance 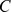 can then be determined by (19). Another possibility is the use of a refractory-density approach [76], [77].

The spike-train correlation as a function of the time lag is an experimentally accessible measure. Future theoretical work should therefore also focus on the temporal structure of correlations in recurrent networks, going beyond zero-lag correlations [15], [17] and the integral measures studied in the current work. This would allow to compare the theoretical predictions to direct experimental observations in a more detailed manner. Moreover, the relative spike timing between pairs of neurons is a decisive property for Hebbian learning [78] in recurrent networks, as implemented by spike timing-dependent plasticity [62], and suspected to play a role for synapse formation and elimination [79].

The simulation experiments performed in this work revealed that the suppression of correlations is vulnerable to certain types of manipulations of the feedback loop. One particular biological source of additional variability in the feedback loop is probabilistic vesicle release at synapses [80]. In feedforward networks, such unreliable synaptic transmission has been shown to decrease the transmission of correlations by pairs of neurons [22]. Stochastic synaptic release is very similar to the replacement of the population activity in the feedback branch by a rate modulated Poisson processes that conserves the population rate. In these simulations we observed an increase of correlations due to the additional noise caused by the stochastic Poisson realization. Future work should investigate more carefully which of the two opposing effects of probabilistic release on correlations dominates in recurrent networks.

The results of our study do not only shed light on the decorrelation of spiking activity in recurrent neural networks. They also demonstrate that a standard modeling approach in theoretical neuroscience is problematic: When studying the dynamics of a local neural network (e.g. a “cortical column”), it is a common strategy to replace external inputs to this neural population 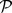 by spike-train ensembles with some predefined statistics, e.g. by stationary Poisson processes. Most neural systems, however, exhibit a high degree of recurrence. Nonlocal input to the population 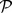, i.e. input from other brain areas, therefore has to be expected to be shaped by the activity within 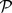. The omission of these feedback loops can lead to qualitatively wrong predictions of the population statistics. The analytical results for the correlation structure of recurrent networks presented in this study provide the means to a more realistic specification of such external activity.

## Methods

### LIF network model

In the present study, we consider two types of sparsely connected random networks: networks with purely inhibitory coupling (“I networks”) and networks with both excitatory and inhibitory interactions (“EI networks”). To illustrate the main findings of this study and to test the predictions of the linear model described in **“** **Methods** **: Linearized network model”**, both architectures were implemented as networks of leaky integrate-and-fire (LIF) neurons. The model details and parameters are reported in Table 1 and Table 2, respectively. All network simulations were carried out with NEST (www.nest-initiative.org, [81]).

**Table 1:** LIF network: Model overview.

A Model summary	
**Populations**	one (inhibitory network) or two (excitatory-inhibitory network)
**Connectivity**	random, fixed in-degrees
**Neuron**	leaky integrate-and-fire (LIF)
**Synapse**	current based, delta-shaped postsyn. currents with constant amplitudes
**Input**	uncorrelated Gaussian white noise currents

**Table 2:** LIF network: Parameters (default values).

A Connectivity		
Name	Value	Description
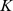	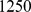 (inhibitory network)	in-degree
	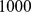 (E-I network)	excitatory in-degree
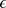	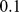	network connectivity
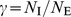	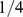 (E-I network)	relative size of inhibitory subpopulation

### Linearized network model

In this section we show how the dynamics of the spiking network can be reduced to an effective linear model with fluctuations fulfilling, by construction, the same relationship as the original system up to linear order. We first outline the conceptual steps of this reduction, and then provide the formal derivation.

We make use of the observation that the effect of a single synaptic impulse on the output activity of a neuron is typically small. Writing the response spike train of a neuron as a functional of the history of all incoming impulses therefore allows us to perform a linearization with respect to each of the afferent spike trains. Formally, this corresponds to a Volterra expansion up to linear order, the generalization of a Taylor series to functionals. In **“** **Methods** **: Response kernel of the LIF model”**, we perform this linearization explicitly for the example of the LIF model. This determines how the linear response kernel depends on the parameters of the LIF model. The linear dependence on the input leads to an approximate convolution equation (31) linearly connecting the auto- and the cross-correlation functions in the network. As this equation is complicated to solve directly, we introduce a reduced linear model (35) obeying the same convolution equation. The reduced linear model can be solved by standard Fourier methods and yields an explicit form for the covariance matrix in the frequency domain (37). The diagonal and off-diagonal elements of the 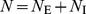 dimensional covariance matrix 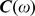 in (56) correspond to the power-spectra of individual neurons and the cross-spectra of individual neuron pairs, respectively. As, in this linear approximation, both the auto- and the cross-covariances are proportional to the variance of the driving noise, the resulting correlation coefficients are independent of the noise amplitude (see **“** **Methods** **: Population averaged correlations in the linear EI network”**). As shown in **“** **Results** **: Suppression of population-activity fluctuations by negative feedback”** and **“** **Results** **: Population-activity fluctuations in excitatory-inhibitory networks”**, the suppression of fluctuations in recurrent networks is most pronounced at low frequencies. It is therefore sufficient to restrict the discussion to the zero-frequency limit 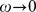. Note that the zero-frequency variances and covariances correspond to the integrals of the auto- and cross-correlation functions in the time domain. In this limit, we may combine the two different sources of fluctuations caused by the spiking of the neurons and by external input to the network into a single source of white noise with variance 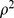 (see (39)).

In general, the spiking activity 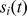 of neuron 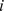 at time 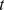 is determined by the entire history 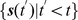 of the activity of all neurons 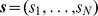 in the network up to time 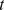. Formally, this dependence can be expressed by a functional


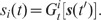
(26)

The subscript 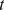 in 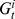 indicates that 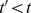 (causality). In the following, we use the abbreviation 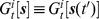. The effect of a single synaptic input on the state of a neuron is typically small. We therefore approximate the influence of an incoming spike train on the activity of the target neuron up to linear order. The sensitivity of neuron 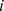
's activity to the input from neuron 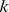 can be expressed by the functional derivative of 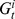 with respect to input spike train 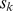:


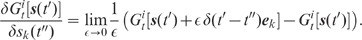
(27)

It represents the response of the functional to a single 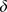-shaped perturbation in input channel 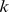 at time 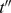, normalized by the perturbation amplitude 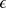. In (27), 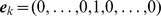 denotes the unity vector with elements 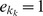 and 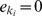 for all 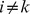. By introducing the vector 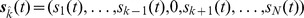 of spike trains with the 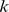-th component set to zero, 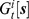 can be approximated by


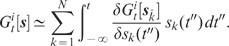
(28)

Eq. (28) is a Volterra expansion up to linear order, the formal extension of a Taylor expansion of a function of 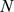 variables to a functional, truncated after the linear term. With the linearized dynamics (28), the pairwise spike-train cross-correlation function between two neurons 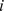 and 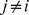 is given by


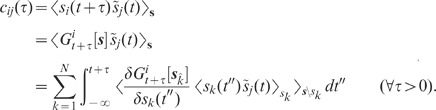
(29)

Note that (29) is valid only for positive time lags 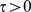, because for 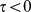 a possible causal influence of 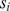 on 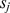 is not expressed by the functional. Here, 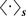 denotes the average across the ensemble of realizations of spike trains in the stationary state of the network (e.g. the ensemble resulting from different initial conditions), and 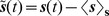 the centralized (zero mean) spike train. In the last line in (29), the average 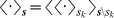 is split into the average 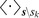 across all realizations of spike trains excluding 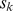 and the average 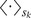 across all realizations of 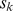. Note that the latter does not affect the functional derivative because it is, by construction, independent of the actual realization of 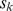. A consistent approximation up to linear order is equivalent to the assumption that for all 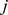 the linear dependence of the functional on 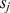 is completely contained in the respective derivative with respect to 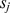 (28). Dependencies beyond linear order include higher-order derivatives and are neglected in this approximation. This is equivalent to neglecting the dependence of 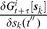 on 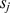 for any 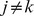. Hence, we can average the inner term over 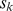 and 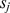 separately. In the stationary state, this correlation can only depend on 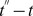 and equals the auto- or the cross-correlation function:


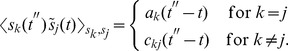


The pairwise spike-train correlation function is therefore given by


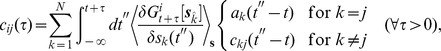


where we used the fact that 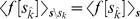 for any functional 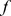 that does not depend on 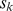. The average of the functional derivative has the intuitive meaning of a response kernel with respect to a 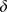-shaped perturbation of input 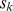 at time 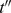. Averaged over the realizations of the stationary network activity this response can only depend on the relative time 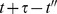. In a homogeneous random network, the input statistics (number of synaptic inputs and synaptic weights) and the parameters of the internal dynamics are identical for each cell, so that the temporal shape 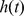 of the response kernel can be assumed to be the same for all neurons. The synaptic coupling strength from neuron 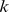 to neuron 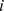 determines the prefactor 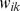:


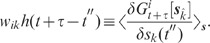
(30)

In this notation, the linear equation connecting the auto-correlations 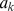 and the cross-correlations 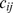 takes the form


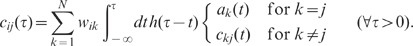
(31)

Eq. (31) can be solved numerically or by means of Wiener-Hopf theory taking the symmetry 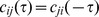 into account [82].

Our aim is to find a simpler model which is equivalent to the LIF dynamics in the sense that it fulfills the same equation (31). Let's 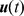 denote the vector of dynamic variables of this reduced model. Analog to the original model, we define the cross-correlation for 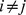 and 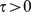 as


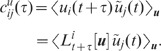
(32)

The simplest functional 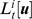 consistent with equation (31) is linear in 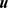. Since we require equivalence only with respect to the ensemble averaged quantities, i.e. 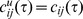, the reduced activity and therefore 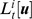 can contain a stochastic element which would disappear after averaging. The linear functional


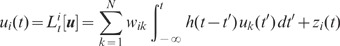
(33)

with a pairwise uncorrelated, centralized white noise 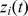 (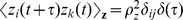) fulfills the requirement, since for 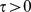 and 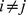



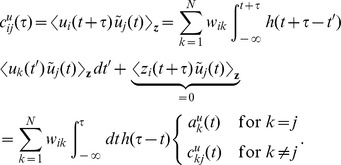


This equation has the same form as (31), so both models, within the linear approximation, exhibit an identical relationship between the auto- and cross-covariances. The physical meaning of the noise 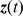 is the variance caused by the spiking of the neurons. The auto-correlation function of a spike train of rate 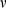 has a 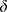-peak of weight 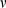. The reduced model (33) exhibits such a 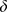-peak if we set 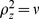. A related approach has been pursued before (see Sec. 3.5 in [31]) to determine the auto-correlation of the population averaged firing rate. This similarity will be discussed in detail below.

So far, we considered a network without external drive, i.e. all spike trains 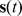 originated from within the network. If the network is driven by external input, each neuron receives, in addition, synaptic input 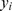 from neurons outside the network. We assume uncorrelated external drive 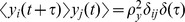. In the reduced model, this input constitutes a separate source of noise:


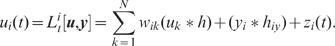
(34)

Here, 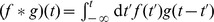 denotes the convolution and 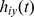 the response kernel with respect to an external input. For simplicity, let's assume that the shape of these kernels is identical for all pairs of pre- and postsynaptic sources, i.e. 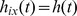. If we further absorb the synaptic amplitude of the external drive in the strength of the noise 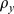, the linearized dynamics (34) can be written in matrix notation


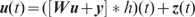
(35)

with 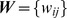. The reduced model (35) can be solved directly by means of Fourier transform:


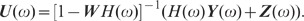
(36)

The full covariance matrix follows by averaging over the sources of noise 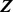 and 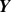 as


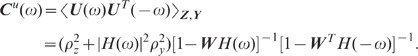
(37)

The diagonal elements of 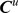 represent the auto-covariances, the off-diagonal elements the cross-covariances. Both are proportional to the driving noise 
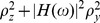
. This is consistent with (31) which is a linear relationship between the cross- and auto-covariances.

For networks which can be decomposed into homogeneous subpopulations, the 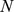 dimensional system (35) can be further simplified by population averaging. Consider, for example, a homogeneous random network with purely inhibitory coupling. Assume that the neurons are randomly connected with probability 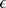 and coupling strength 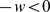. The average number of in/outputs per neuron (in/out-degree) is thus given by 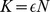. By introducing the population averaged external input 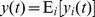, the averaged spiking noise 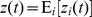, and the effective coupling strength 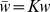, the dynamics of the population averaged activity becomes


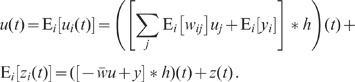
(38)

Here we assumed that 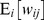 is independent of the presynaptic neuron 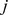 and can be replaced by 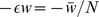. Note that this replacement is exact for networks with homogeneous out-degree, i.e. if the number of outgoing connections is identical for each neuron 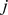. For large random networks with binomially distributed out-degrees (e.g. Erdös-Rényi networks or random networks with constant in-degree), (38) serves as an approximation.

To relate our approach to the treatment of finite-size fluctuations in [31], consider the population-averaged dynamics (38) of a single population with mean firing rate 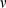. We set 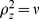 for all single neuron noises 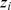 in order for the reduced model's auto-covariances to reproduce the 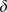-peak of the spiking dynamics. In the population averaged dynamics, this leads to the variance of the noise 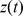 given by 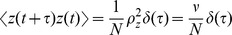. This agrees with the variance of the population rate in [31]. Therefore, the dynamics of the population averaged quantity 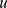 in (38) agrees with the earlier definition of a population averaged firing rate 
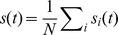
 for the spiking network [31].

In equation (38), two distinct sources of noise appear: The noise due to external uncorrelated activity 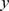 and the noise 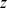 which is required to obtain the 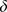-peak of the auto-correlation functions of the reduced model. The qualitative results of **“** **Results** **: Suppression of population-activity fluctuations by negative feedback”** and **“** **Results** **: Population-activity fluctuations in excitatory-inhibitory networks”**, however can be understood with an even simpler model. As we are mainly concerned with the low-frequency fluctuations, we only need a model with the same limit 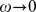. As we normalized the kernel so that 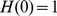 we can combine both sources of noise and require 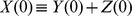 in (36) in the zero frequency limit. Hence, in **“** **Results** **: Suppression of population-activity fluctuations by negative feedback”** and **“** **Results** **: Population-activity fluctuations in excitatory-inhibitory networks”**, we consider the model


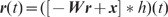
(39)

with a pairwise uncorrelated centralized white noise 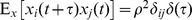 to explain the suppression of fluctuations at low frequencies.

As a second example, consider a random network composed of an excitatory and an inhibitory subpopulation 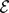 and 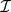 with population sizes 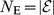 and 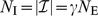, respectively. Assume that each neuron receives excitatory and inhibitory inputs from 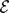 and 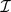 with coupling strengths 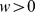 and 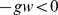, respectively, and probability 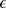, such that the average excitatory and inhibitory in/out-degrees are given by 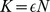 and 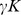, respectively. The dynamics of the subpopulation averaged activities 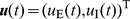 is given by (35) with subpopulation averaged noise 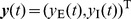 and 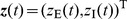 and effective coupling


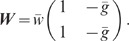
(40)

Here, 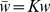 denotes the effective coupling strength, 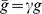 the effective balance parameter and 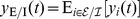 and 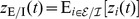 the (sub)population averaged external and spiking sources of noise, respectively. Again, the reduction of the 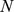-dimensional linear dynamics to the two-dimensional dynamics (40) is exact if the out-degrees are constant within each subpopulation. As before, both sources of noise can be combined into a single source of noise, if we are only interested in the low-frequency behavior of the model, leading to the dynamics (39) with the effective coupling (40).

The linear theory is only valid in the domain of its stability, which is determined by the eigenvalue spectrum of the effective coupling matrix 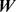. For random coupling matrices, the eigenvalues are located within a circle with a radius equal to the square root of the variance of the matrix entries [69] 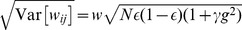. Writing the effective dynamics for the exponential kernel as a differential equation 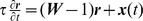, the eigenvalues of the right hand side matrix 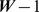 are confined to a circle centered at 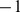 in the complex plain with radius 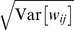. Given 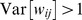, eigenvalues might exist which have a positive real part, leading to unstable dynamics. This condition is indicated by the vertical dotted lines in Fig. 6 A–F and Fig. 7 B–D near 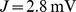. Beyond this line, the linear model predicts an explosive growth of fluctuations. In the LIF-network model, an unbounded growth is avoided by the nonlinearities of the single-neuron dynamics.

### Response kernel of the LIF model

We now perform the formal linearization (30) for a network of 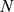 LIF neurons 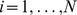. A similar approach has been employed in previous studies to understand the population dynamics in these networks [12], [31]. We consider the input 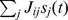 received by neuron 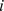 from the local network, where 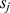 denotes the spike train of the neuron 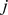 projecting to neuron 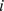 with synaptic weight 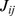. Given the time dependent firing rate 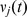 of each afferent, and assuming small correlations and small synaptic weights, the total input to neuron 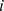 can be replaced by a Gaussian white noise with mean 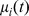 and variance 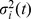,


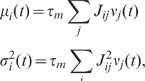
(41)

where 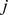 sums over all synaptic inputs. 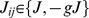 denotes the amplitude of the postsynaptic potential evoked by synapse 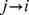. 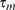 is the membrane time constant of the model. In the stationary state, the firing rate of each afferent is well described by the constant time average 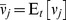. The working point at which we perform the linearization of the neural response (30) is then given by analog equations as (41), resulting in a constant mean 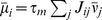 and variance 
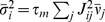
. If the amplitude of each postsynaptic potential is small compared to the distance of the membrane potential to threshold, the dynamics of the LIF model can be approximated by a diffusion process, employing Fokker-Planck theory [83]. The stationary firing rate of the neuron is then given by [12], [31], [84]


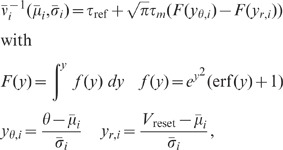
(42)

with the reset voltage 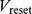, the threshold voltage 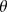 and the refractory time 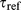. In homogeneous random networks, the stationary rate (Fig. 7 A) is the same for all neurons. It is determined in a self-consistent manner [12] as the fixed point of (42). The stationary mean 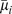 and variance 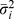 are determined by the stationary rate. To determine the kernel (30) we need to consider how a 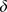-shaped deflection in the input to this neuron at time point 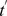 affects its output up to linear order in the amplitude of the fluctuation. In the stationary state, we may set 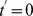. It is therefore sufficient to focus on the effect of a single fluctuation


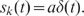
(43)

We therefore ask how the density of spikes per time 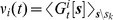 of neuron 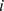, averaged over different realizations of the remaining inputs to neuron 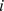, changes in response to the fluctuation (43) of the presynaptic neuron 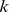 in the limit of vanishing amplitude 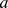. This kernel 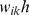 (30) is identical to the impulse response of the neuron and can directly be measured in simulation by trial averaging over many responses to the given 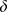-deflection (43) in the input (see Fig. 8 A). For the theory of low-frequency fluctuations, we only need the integral of the kernel, also known as the DC susceptibility,


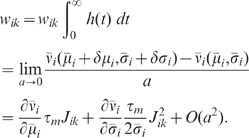
(44)

The second equality follows from the equivalence of the integral of the impulse response and the step response in linear approximation [21], [33]. Following from [41], both mean and variance are perturbed as 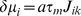 and 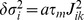 in response to a step 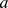 in the afferent rate 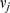. Moreover, we used the chain rule 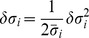. The variation of the afferent firing rate hence co-modulates the mean and the variance and both modulations need to be taken into account to derive the neural response [31]. Although the finite amplitude of postsynaptic potentials has an effect on the response properties [33], [34], the integral response is rather insensitive to the granularity of the noise [33]. We therefore employ the diffusion approximation to linearize the dynamics of the LIF neuron around its working point characterized by the mean 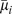 and the variance 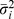 of the total synaptic input. In (44), we evaluate the partial derivatives of 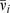 with respect to 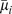 and 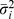 using (42). First, observe that by chain rule 
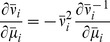
. We then again make use of the chain rule 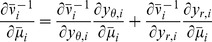. Analog expressions hold for the derivative with respect to 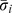. The first derivative yields 
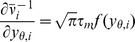
, the one with respect to 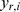 follows analogously, but with a negative sign. We further observe that 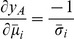 and 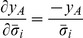 with 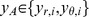. Taken together, we obtain the explicit result for (44)


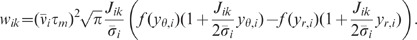
(45)

Note that the modulation of 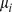 results in a contribution to 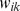 that is linear in 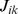, whereas the modulation of 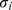 causes a quadratic dependence on 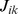. This expression therefore presents an extension to the integral response presented in [21], [85]. Fig. 8 B shows the comparison of the analytical expression (45) and direct simulation. The agreement is good over a large range of synaptic amplitudes 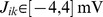 in the case of constant background noise caused by small synaptic amplitudes (here 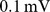 for excitation and 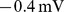 for inhibition). For background noise caused by stronger impulses, the deviations are expected to grow [33].

### Population-activity spectra in the linear model: feedback vs. feedforward scenario

The recurrent linear neural dynamics defined in the previous section is conveniently solved in the Fourier domain. The driving external Gaussian white noise 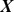 is mapped to the response 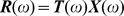 by means of the transfer matrix 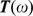. According to (39), it is given by 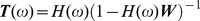. The covariance matrix in the frequency domain, the spectral matrix, thus reads


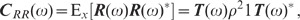
(46)

where we used 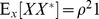 and the expectation operator 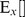 represents an average over noise realizations. To identify the effect of recurrence on the network dynamics, we replace the local feedback input by a feedforward input 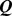 with spectral matrix 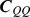. The resulting response firing rate is given by 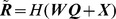. Assuming that the feedforward input 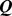 is uncorrelated to the external noise source 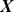 (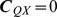) yields a response spectrum


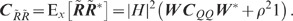
(47)

### Population-activity spectrum of the linear inhibitory network

In the Fourier domain, the solution of the mean-field dynamics (38) of the inhibitory network is 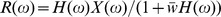. The power-spectrum 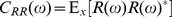 hence becomes


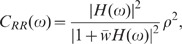
(48)

using the spectrum of the noise 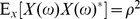.

We compare this power-spectrum to the case where the feedback loop is opened, i.e. where the recurrent input is replaced by feedforward input with unchanged auto-statistics 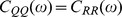, but which is uncorrelated to the external input 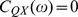. The resulting power-spectrum is given by (47) as 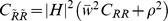.

### Population-activity spectra of the linear excitatory-inhibitory network

In a homogeneous random network of excitatory and inhibitory neurons, the population averaged activity (40) can be solved in the Schur basis (9) introduced in **“** **Results** **: Population-activity fluctuations in excitatory-inhibitory networks”**


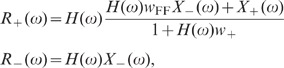
(49)

with 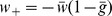 and 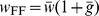. The power of the population rate therefore is


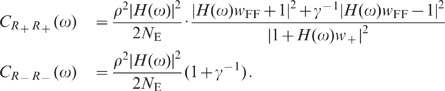
(50)

The fluctuations of the excitatory and the inhibitory population follow as


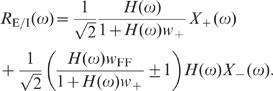
(51)

So the power-spectra are


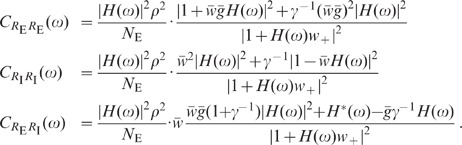
(52)

Replacing the recurrent input of the sum activity 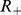 by activity 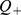 with the same auto-statistics, but which is uncorrelated to the remaining input into 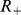 (Fig. 5 D′) results in the fluctuations


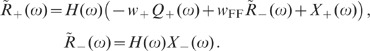
(53)

The power-spectrum of the sum activity therefore becomes


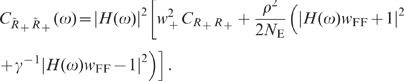
(54)

If, alternatively, the excitatory and the inhibitory feedback terms 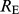 and 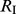 are replaced by uncorrelated feedforward input 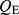 and 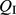 with power-spectra 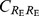 and 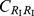 (Fig. 5 C,D), the spectrum of the sum activity reads


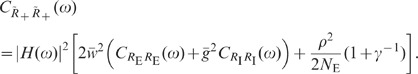
(55)

The limit (14) for inhibition dominated networks with 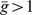 can be obtained from this and the former expressions by taking 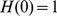 and assuming strong coupling 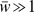.

### Population averaged correlations in the linear EI network

In this subsection, we derive a self-consistency equation for the covariances in a recurrent network. We start from (37) (we drop the superscript 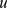 of 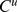 for brevity) multiply by 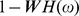 from left and its transpose from right to obtain


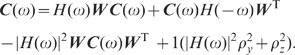
(56)

We assume a recurrent network of 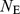 excitatory and 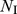 inhibitory neurons, in which each neuron receives 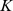 excitatory inputs of weight 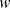 and 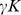 inhibitory inputs of weight 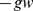 drawn randomly from the presynaptic pool of neurons. To obtain a theory for the variances and covariances at zero frequency (with 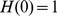) we may abbreviate 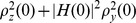 by 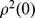. For a population averaged theory, we need to replace in (56) the variances 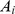 of an individual neuron by the population average and replace the covariance 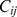 for a given pair of neurons 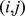 by the average over pairs that are statistically equivalent to 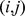. For a pair 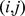 of neurons we will show that the set of equivalent pairs depends on the current realization of the connectivity since unconnected pairs are not equivalent to connected ones. Therefore it is necessary to first average the covariance matrix over statistically equivalent neuron pairs given a fixed connectivity and to subsequently average over all possible realizations of the connectivity. The latter will be denoted as 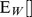. For compactness of the notation, first we perform the averaging for the general case, where neuron 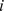 belongs to population 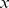 and neuron 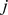 to population 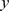. We denote by 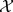, 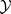 the sets of neuron indices belonging to populations 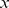 and 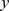, respectively. Subsequently replacing 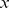 and 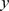 by all possible combinations 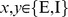, we obtain the averaged self-consistency equations for the network. We denote the number of incoming connections to a neuron of type 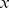 from the population of neurons of type 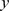 as 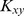 and the strength of a synaptic coupling as 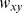. Rewriting the self-consistency equation (56) explicitly with indices yields


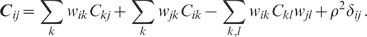
(57)

The last equation shows that for a connected pair 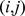 of neurons (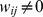 or 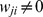) either of the first two sums contains a contribution 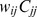 or 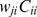 proportional to the variance of the projecting neuron. We therefore need to perform the averaging separately for connected and for unconnected pairs of neurons. We use the notation


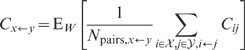
(58)

for the average covariance over pairs of neurons of types 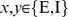 with a connection from neuron 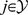 to neuron 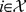, where 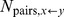 is the number neuron pairs connected in this way. An arrow to the right, 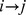, denotes a connection from neuron 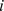 to neuron 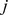. Note that we use the same letter 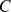 for the population averaged covariances and for the covariances of individual pairs. The distinction can be made by the indices: 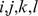 throughout indexes a single neuron, 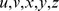 identifies one of the populations 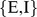. We denote the covariance averaged over unconnected pairs as


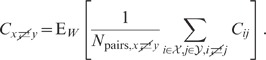
(59)

We further use


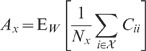
(60)

for the integrated variance averaged over all neurons of type 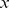. Connected and the unconnected averaged covariances differ by the term proportional to the variance of the projecting neuron, as mentioned above


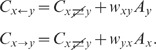
(61)

As a consequence, we can express all quantities in terms of the averaged variance (60) and the covariance averaged over unconnected pairs (59). We now proceed to average the integrated variance over population 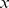. Since there are no self-connections in the network, we do not need to distinguish two cases here. Replacing 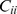 on the right hand side of (60), the first term of (57) contributes


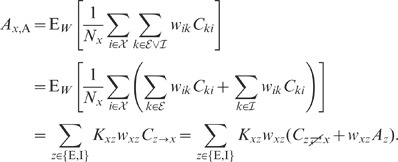
(62)

From the second to the third step we used that the sum over 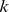 (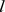) yields non-zero contributions only if neuron 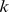 (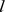) connects to neuron 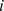. This happens in 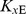 (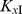) cases with the coupling weight 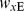 (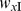). Therefore the covariance averaged over connected pairs appears on the right hand side. In the last line we used the relation (61) to express the connected covariance in terms of the variance and the covariance over unconnected pairs. The second term in (60) is identical because of the symmetry 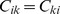. Up to here, the structure of the network only entered in terms of the in-degree of the neurons. The contribution of the third term follows from a similar calculation


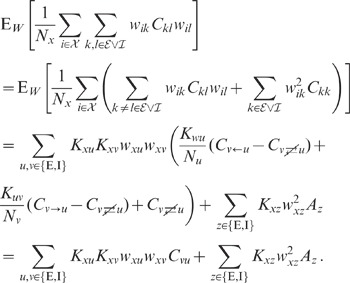
(63)

From the second to the third step we assumed that among the 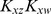 pairs of neurons 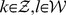 projecting to neuron 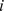, the fraction 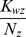 has a connection 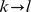. These pairs contribute with the connected covariance. The connections in opposite direction contribute the other term of similar structure. We ignore multiple and reciprocal connections here, assuming the connection probability is low. We introduce the shorthand 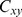 for the covariance averaged over all neuron pairs including connected and unconnected pairs


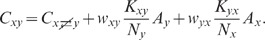
(64)

This is the covariance which is observed on average when picking a pair of neurons of type 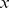 and 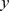 randomly. In this step, beyond the in-degree, the structure of the network entered through the expected number of connections between two populations. Taken all three terms together, we arrive at


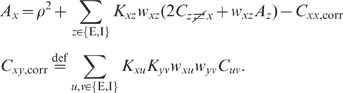
(65)

The averaged covariances follow by similar calculations. Here we only need to calculate the average over unconnected pairs 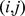 given by (59), because the connected covariance follows from (61). The first sum in (57) contributes


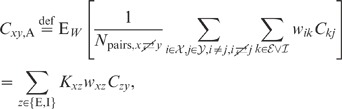
(66)

where due to the absence of a direct connection between 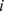 and 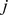, the term linear in the coupling and proportional to the variance is absent. From the symmetry 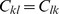 it follows that the second term corresponds to an exchange of 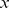 and 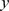 in the last expression. The third sum in (57) follows from an analog calculation as before


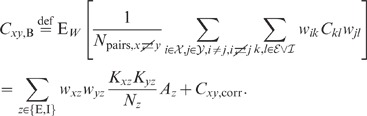
(67)

In summary, the contributions from (66) and (67) together result in the self-consistency equation for the covariance


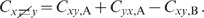
(68)

We now simplify the expressions by assuming that the in-degree of a neuron and the incoming synaptic amplitudes do not depend on the type of the neuron, i.e. that excitatory and inhibitory neurons receive statistically the same input. Formally this means that we need to replace 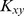 by 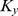, the number of incoming connections from population 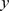 and 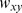 by 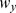, the coupling strength of a projection from a neuron of type 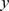. The covariance 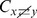 then has two distinct contributions, 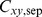 that depends on the type of neurons 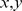, and 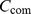 that does not. In particular 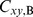 and 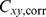 do not depend on 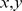 and we omit their subscripts in the following. The variances fulfill


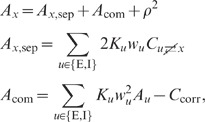
(69)

the covariances satisfy


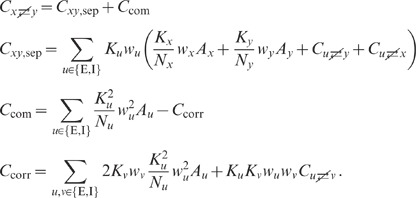
(70)

The disjoint part 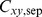 determines the difference between the covariances for pairs of neurons of different type. Using the parameters 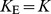, 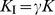, 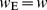, 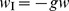, the explicit form is


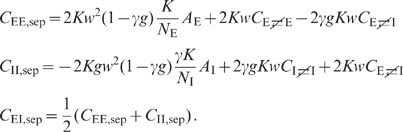
(71)

Therefore, also the covariances in the network obey the relation


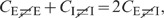
(72)

i.e. the mixed covariance can be eliminated and is given by the arithmetic mean of the covariances between neurons of same type. In matrix representation with the vector 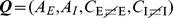, the self-consistency equation is


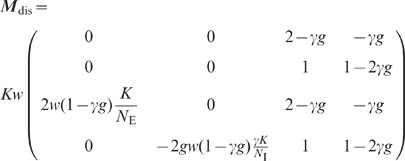
(73)


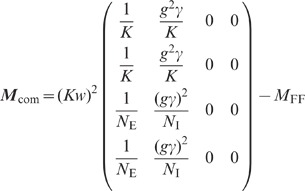
(74)


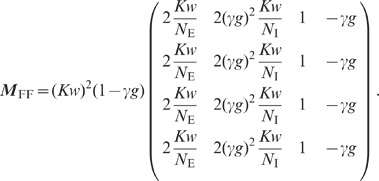
(75)

The self consistent covariance can then be obtained by solving the system of linear equations


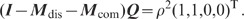
(76)

The numerical solution shows that the variances for excitatory and inhibitory neurons are approximately the same, as depicted in Fig. 6 A. In the following we therefore assume 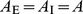 and then solve (76) for the covariances. With the abbreviation 
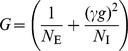
, the third and fourth line yields the equation for the covariances


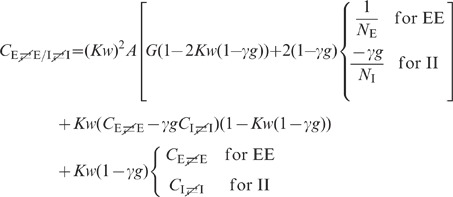
(77)

The structure of the equation suggests to introduce the linear combination 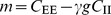 which satisfies


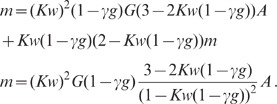
(78)

We solve (77) for 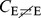 and 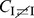 and insert (78) for 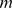 to obtain the covariances as


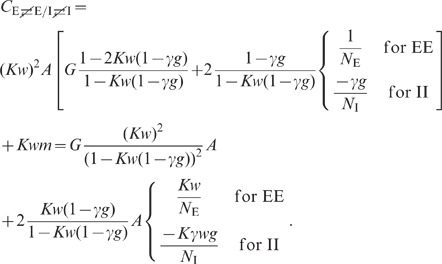
(79)

The covariance 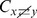 between unconnected neurons can be related to the covariance between the incoming currents this pair of neurons receives. Expressing the self-consistency (68) in terms of the covariances averaged over connected and unconnected pairs (64) uncovers the connection


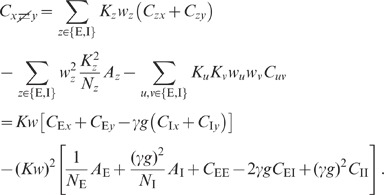
(80)

This self-consistency equation yields the argument, why the shared-input correlation 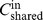 (19) cancels the contribution 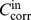 (20) due to spike-train correlations in the covariance to the input currents (see Fig. 6 C,D). Rewriting (80) in terms of these quantities results in


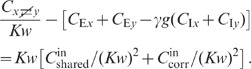
(81)

If a self-consistent solution with small correlation 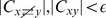 exists, the right hand side of (81) must be of the same order of magnitude. The right hand side of this equation has a prefactor 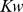 which typically is 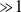 (for the parameters in Fig. 6, 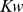 becomes larger than 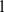 for 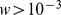). The first term in the bracket is proportional to the contribution of shared input, the second term is due to correlations among pairs of different neurons. Each of these terms is of order 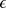. Due to the prefactor 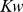, however, the sum of the two terms needs to be of order 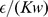 to fulfill the equation. Hence, the terms must have different signs to cause the mutual cancellation.

To illustrate how the correlation structure is affected by feedback, let us now consider the case where the feedback activity is perturbed (“feedforward scenario”). We start from (47) and, again, only consider the fluctuations at zero frequency,


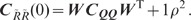
(82)

First, we consider a manipulation that preserves the single-neuron statistics 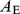, 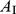 and the pairwise correlations 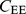, 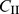 within each subpopulation, but neglects correlations 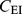 between excitatory and inhibitory neurons. Formally, this corresponds to the block diagonal correlation matrix


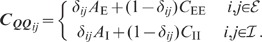
(83)

Here, we have replaced the individual entries of the correlation matrix by the corresponding subpopulation averaged correlations. The calculation of the response auto- and cross-correlation 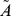 and 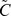 is similar as for the expressions (63) and (67), with the difference that terms containing 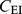 are absent:


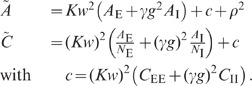
(84)

As an alternative type of feedback manipulation, we assume that all correlations are equal, irrespective of the neuron type. To this end, we replace all spike correlations by the population average 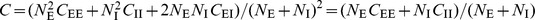. Thus, the covariance matrix reads


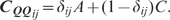
(85)

The calculation follows the one leading to the expressions (63) and (67) and results in


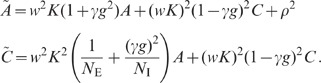
(86)
